# Fine-tuned local coordination environment of Pt single atoms on ceria controls catalytic reactivity

**DOI:** 10.1038/s41467-022-34797-2

**Published:** 2022-11-18

**Authors:** Wei Tan, Shaohua Xie, Duy Le, Weijian Diao, Meiyu Wang, Ke-Bin Low, Dave Austin, Sampyo Hong, Fei Gao, Lin Dong, Lu Ma, Steven N. Ehrlich, Talat S. Rahman, Fudong Liu

**Affiliations:** 1 Department of Civil, Environmental, and Construction Engineering, Catalysis Cluster for Renewable Energy and Chemical Transformations (REACT), NanoScience Technology Center (NSTC), University of Central Florida, Orlando, FL 32816 USA; 2 State Key Laboratory of Pollution Control and Resource Reuse, School of the Environment; Jiangsu Key Laboratory of Vehicle Emissions Control, School of Chemistry and Chemical Engineering; Center of Modern Analysis, Nanjing University, Nanjing, 210023 China; 3 Department of Physics, Catalysis Cluster for Renewable Energy and Chemical Transformations (REACT), University of Central Florida, Orlando, FL 32816 USA; 4 Department of Chemical and Biological Engineering, Villanova University, Villanova, PA 19085 USA; 5 College of Engineering and Applied Sciences, Nanjing University, Nanjing, 210093 China; 6 BASF Corporation, Iselin, NJ 08830 USA; 7 Brewton-Parker College, Mount Vernon, GA 30445 USA; 8 National Synchrotron Light Source II (NSLS-II), Brookhaven National Laboratory, Upton, NY 11973 USA

**Keywords:** Pollution remediation, Atmospheric chemistry, Heterogeneous catalysis

## Abstract

Constructing single atom catalysts with fine-tuned coordination environments can be a promising strategy to achieve satisfactory catalytic performance. Herein, via a simple calcination temperature-control strategy, CeO_2_ supported Pt single atom catalysts with precisely controlled coordination environments are successfully fabricated. The joint experimental and theoretical analysis reveals that the Pt single atoms on Pt_1_/CeO_2_ prepared at 550 °C (Pt/CeO_2_-550) are mainly located at the edge sites of CeO_2_ with a Pt–O coordination number of *ca*. 5, while those prepared at 800 °C (Pt/CeO_2_-800) are predominantly located at distorted Ce substitution sites on CeO_2_ terrace with a Pt–O coordination number of *ca*. 4. Pt/CeO_2_-550 and Pt/CeO_2_-800 with different Pt_1_-CeO_2_ coordination environments exhibit a reversal of activity trend in CO oxidation and NH_3_ oxidation due to their different privileges in reactants activation and H_2_O desorption, suggesting that the catalytic performance of Pt single atom catalysts in different target reactions can be maximized by optimizing their local coordination structures.

## Abstract (web summary)

CeO_2_-supported Pt single atoms with different coordination environments exhibit distinct behaviors in reactant adsorption/activation and product desorption, thus showing different catalytic performance for various catalytic oxidation reactions.

## Introduction

In recent years, different types of single atom catalysts have been applied in many energy and environment-related reactions because of their maximum atomic utilization efficiency, unique electronic states, or higher stability comparing to the nano-cluster/particle catalysts^1–7^. With the deepening of research in this area, it has been revealed that the performance of single atom catalysts on specified supports for certain reactions such as thermal catalysis and electrocatalysis could be significantly promoted by tuning their oxidation states or coordination environments^8–14^. Among these single atom catalytic materials, the Pt single atom (Pt_1_) catalyst supported on rare earth metal oxide CeO_2_ is one of the most popular catalyst systems, which has been studied extensively for the elimination of environmental pollutants and energy conversion^15–19^.

Most recently, various synthesis methods have been employed to fabricate Pt single atoms with improved catalytic performance^18–20^. For instance, by creating stable hydroxyl groups on Pt_1_/CeO_2_ catalyst using hydrothermal treatment at 750 °C, the CO oxidation activity on Pt_1_/CeO_2_ could be enhanced dramatically^20^. Ma et al. reported that modulating Pt_1_/CeO_2_ with phosphate (PO_4_^3−^) could increase the valence state of Pt and facilitate the reactant adsorption and hydrogen spillover, which accordingly boosted the catalytic activity for styrene hydrogenation^21^. Jeong et al. tuned the oxidation state of Pt single atoms on CeO_2_ using hydrogen reduction method for maximizing the catalytic activity in CO, CH_4_, and NO oxidation^22^. Meanwhile, many experimental and theoretical efforts have been devoted to synthesizing Pt_1_ catalysts on CeO_2_ support with optimal coordination environment or constructing reasonable Pt_1_–CeO_2_ structural models, which could help better understand the reaction mechanisms and reveal the origin of different catalytic performances on Pt single atom catalysts^23–28^. For example, Jiang et al. successfully synthesized a Pt_1_/CeO_2_ catalyst with asymmetric Pt_1_–O_4_ configuration, which exhibited much higher CO oxidation activity than the reference catalyst with symmetric Pt_1_–O_4_ configuration^27^. Wang et al. reported that CO–Pt_1_–O_3_ was the dominant configuration for CO adsorption on Pt_1_ catalyst supported on oxygen plasma-pretreated CeO_2_, and such Pt_1_ species exhibited higher CO oxidation activity and better resistance towards sintering comparing to that in conventional Pt_1_/CeO_2_ catalyst^28^. So far, no comprehensive work has been reported focusing on the precise tuning of local coordination environment for Pt single atoms on CeO_2_ support to modulate their activity in different catalytic reactions and at the same time revealing the intrinsic structure-activity relationships. Herein, we proposed a simple strategy for fine-tuning the exact location of Pt single atoms by controlling the calcination temperature of Pt/CeO_2_ catalysts prepared by the facile incipient wetness impregnation (IWI) method. The Pt single atoms on Pt_1_/CeO_2_ catalysts calcined at different temperatures could have different coordination environments. Through systematic experimental characterizations, catalytic performance testing, and density functional theory (DFT) based simulations of Pt_1_/CeO_2_ catalysts, it was clearly revealed that the CeO_2_-supported Pt single atoms with fine-tuned local coordination structures could exhibit strikingly distinct catalytic behaviors in different oxidation reactions such as CO oxidation versus NH_3_ oxidation, which needs to be taken into consideration for practical applications of single atom catalysis in the future.

## Results

### Constructing Pt_1_ with different coordination environments

To minimize the potential structural change of pure CeO_2_ support after the deposition of Pt followed by calcination, the CeO_2_ used in this work was pre-calcined in air at 800 °C for 12 h to obtain pre-stabilized structure. Moreover, the deposition of Pt was reported to have a positive effect on stabilizing the CeO_2_ support^29^. As shown in Supplementary Fig. 1 and Table 1, the pure CeO_2_ support and CeO_2_ supported Pt catalysts showed almost the same specific surface area and pore structure, suggesting no obvious change in the textural structure of CeO_2_ support during the catalyst fabrication process.

**Table 1:** Textural properties determined by N_2_ physisorption results, band gap determined by UV–Vis spectra, and the average valence states of Pt species determined by XANES linear combination fitting

Samples	*S*_BET_ (m^2^/g)	Pore volume (cm^3^/g)^a^	Band gap (eV)^b^	Average valence state^c^
CeO_2_	62.5	19.2	3.23	–
Pt/CeO_2_-350	58.3	18.4	3.00	3.6 ± 0.1
Pt/CeO_2_-550	59.5	16.4	2.96	3.4 ± 0.1
Pt/CeO_2_-700	57.9	18.0	2.80	2.9 ± 0.1
Pt/CeO_2_-800	59.5	17.2	2.61	2.7 ± 0.1

^a^The pore volume was determined by BJH method using desorption isotherms.

^b^The band gap was calculated by the intersection of the extrapolated linear portions on plots of (*αhv*)^2^ vs. photon energy (*hv*) to the zero absorbance.

^c^The average valence states of Pt species were obtained from the linear combination fitting analysis of Pt L_3_-edge XANES.

**Fig. 1:**
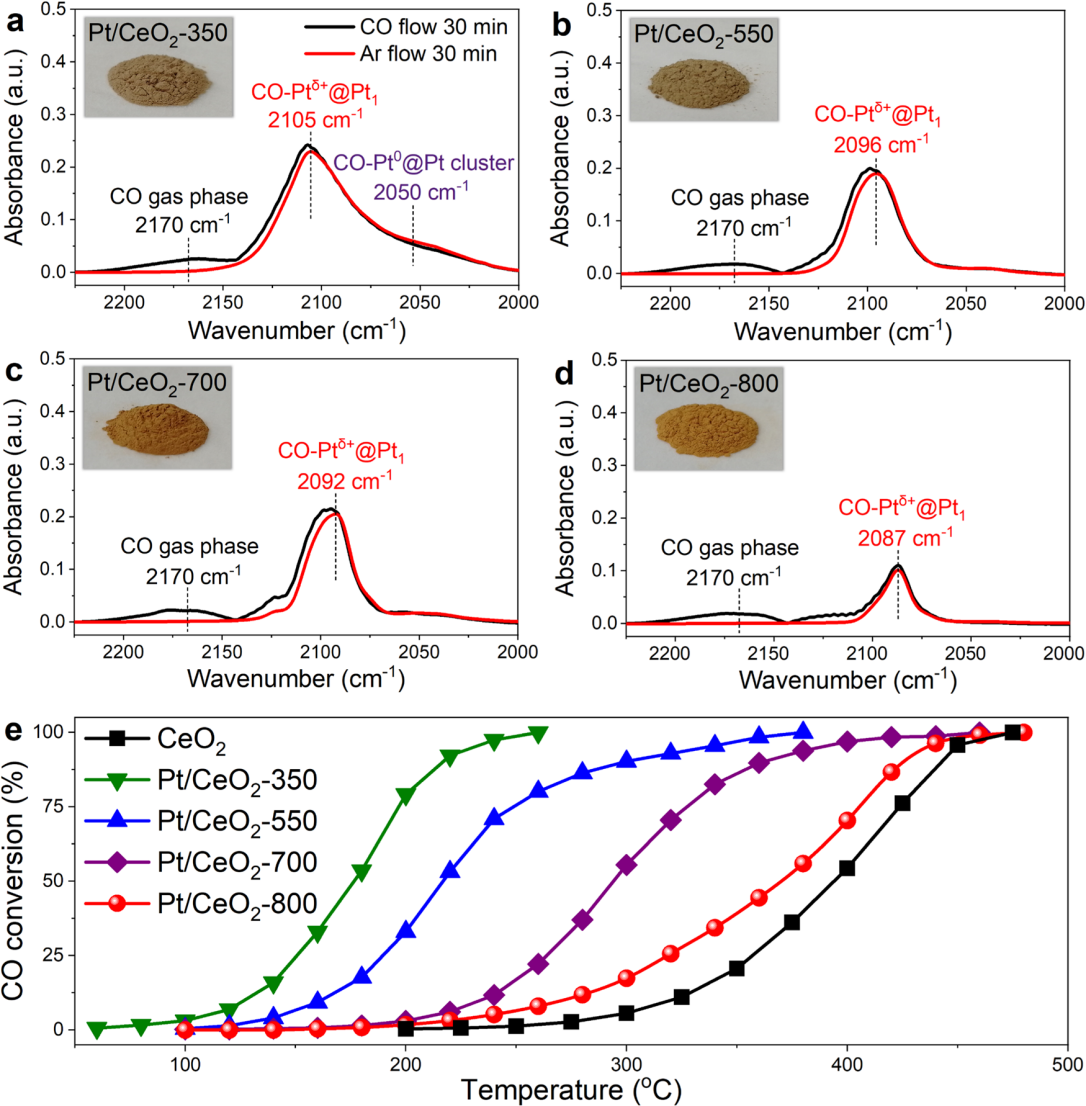
Pt status determined by CO adsorption and corresponding CO oxidation activity. In situ DRIFTS of CO adsorption at 25 °C on **a** Pt/CeO_2_-350, **b** Pt/CeO_2_-550, **c** Pt/CeO_2_-700 and **d** Pt/CeO_2_-800 (inserted are the photos of different catalysts); **e** CO oxidation performance on Pt/CeO_2_-350, Pt/CeO_2_-550, Pt/CeO_2_-700 and Pt/CeO_2_-800 (Reaction condition: [CO] = [O_2_] = 1%, balanced with Ar, WHSV = 400,000 mL∙g_cat_^−1^∙h^−1^).

To determine the states of Pt species on Pt/CeO_2_ catalysts, in situ DRIFTS of CO adsorption at 25 °C was first performed (Fig. 1). The IR bands assigned to CO adsorbed on ionic Pt single sites (CO-Pt^δ+^@Pt_1_) were observed on all four catalysts at 2087–2105 cm^−1^. For Pt/CeO_2_-350, a broad band at *ca*. 2050 cm^−1^ ascribed to CO adsorbed on metallic Pt sites from Pt clusters (CO-Pt^0^@Pt clusters) was also detected. Although the Pt species on Pt/CeO_2_-550, Pt/CeO_2_-700, and Pt/CeO_2_-800 were mainly in the form of single atoms, the monotonical redshift of CO-Pt^δ+^@Pt_1_ band as the calcination temperature increased indicated the different local coordination environments or oxidation states of Pt single atoms. As concluded from previous works, the center of the IR band assigned to CO linearly adsorbed on Pt_1_ supported by CeO_2_ usually ranged between 2085 and 2105 cm^−1^^24,27,30,31^. The 9 cm^−1^ redshift of IR band (from 2096 to 2087 cm^−1^) for CO adsorption as the calcination temperature increased from 550 to 800 °C could be considered as a significant change, indicating the remarkably distinct Pt_1_ local structures on Pt/CeO_2_-550 and Pt/CeO_2_-800. Moreover, the Pt/CeO_2_ catalysts calcined at different temperatures showed varied colors (from dark brown to golden brown), suggesting the different states of Pt_1_ species as well (inserted photos in Fig. 1a–d).

CO oxidation is a commonly used probe reaction to evaluate the catalytic oxidation performance of Pt–CeO_2_-based catalysts and many other catalyst systems^32–34^. In turn, the difference in the local structure of Pt–CeO_2_ catalysts can be reflected by their corresponding CO oxidation activity^27,35^. As demonstrated in Fig. 1e, with the increase of calcination temperature, the CO oxidation activity on Pt/CeO_2_ catalysts decreased monotonically. The presence of Pt clusters on Pt/CeO_2_-350 (Fig. 1a) well explained its best CO oxidation activity, as the Pt cluster-CeO_2_ interfacial sites were considered as the most active species for CO oxidation^30,36–38^. Although the Pt species on Pt/CeO_2_-550, Pt/CeO_2_-700, and Pt/CeO_2_-800 were all in Pt single atom form, the significant difference in the CO oxidation activity on Pt/CeO_2_-550, Pt/CeO_2_-700, and Pt/CeO_2_-800 revealed that the Pt single atoms with varied states were successfully created on these three catalysts.

**Fig. 2:**
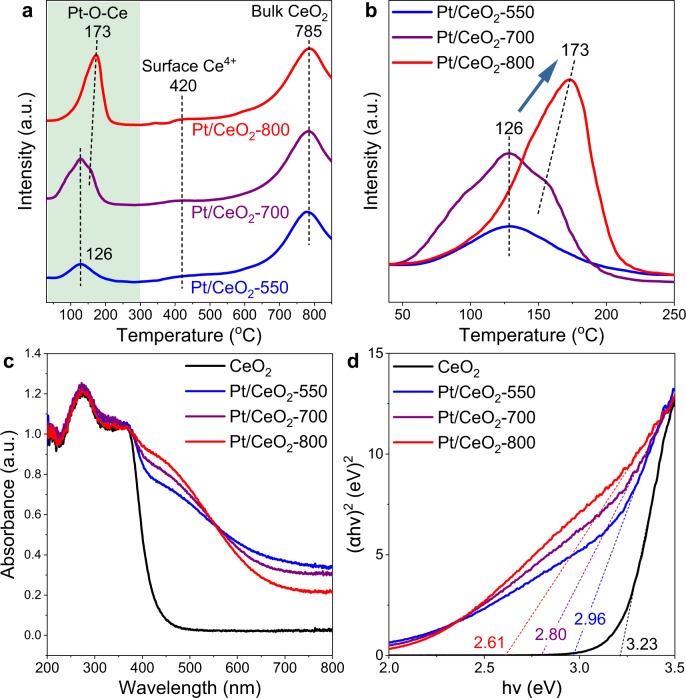
Pt-CeO_2_ interaction determined by H_2_-TPR and UV–Vis spectra. **a**, **b** H_2_-TPR profiles for Pt/CeO_2_-X catalysts; **c** UV–Vis spectra of CeO_2_ support and Pt/CeO_2_-X catalysts; **d** the plots of (*αhv*)^2^ vs. photon energy (*hv*) for CeO_2_ support and Pt/CeO_2_-X catalysts (X = 550, 700 and 800).

H_2_-TPR technique is a powerful tool to evaluate the dispersion of Pt species as well as the strength of Pt-O-Ce interaction on Pt/CeO_2_ based catalysts^39,40^. Herein, the H_2_-TPR profiles of Pt/CeO_2_-550, Pt/CeO_2_-700, Pt/CeO_2_-800 were collected to investigate the interaction between Pt single atoms and CeO_2_ support (Fig. 2a, b). The H_2_-consumption peaks centered around 150 °C could be attributed to the reduction of Pt–O–Ce structure, while the H_2_-consumption peaks at *ca*. 420 and 785 °C could be ascribed to the reduction of surface Ce^4+^ species and bulk CeO_2_, respectively^41^. Interestingly, as the calcination temperature increased from 550 to 800 °C, the H_2_-consumption peak assigned to the reduction of Pt–O–Ce structure shifted to higher temperatures (126 °C → 173 °C) with enhanced intensity, indicating the different strength of Pt–O–Ce interaction as well as the different locations of Pt single atoms on Pt/CeO_2_-X catalysts. For Pt/CeO_2_-700, the Pt singe atoms should be in a mixed state of the Pt species in Pt/CeO_2_-550 and Pt/CeO_2_-800. Based on the CO oxidation evaluation results, Pt/CeO_2_-800 catalyst showed very similar CO oxidation activity to the pure CeO_2_ support. Therefore, the much more intensive H_2_-consumption peak of Pt–O–Ce species at higher reduction temperature on Pt/CeO_2_-800 suggested that the Pt single atoms on this catalyst might have migrated into the surface lattice of CeO_2_ support and the isolated Pt atoms were strongly bonded to CeO_2_ through more Pt–O–Ce linkages.

UV–Vis spectroscopy analysis was also conducted to study the interaction between Pt single atoms and CeO_2_ support. As shown in Fig. 2c, the band below 400 nm on CeO_2_ and Pt/CeO_2_-X catalysts (X = 550, 700, and 800) could be attributed to the charge transfer for O_*2p*_ → Ce_*4f*_ transition^42^. Interestingly, after the deposition of Pt, an additional strong absorption assigned to the absorption tail or Urbach tail was observed at 400–600 nm, which was related to the band gap smearing induced by defect accumulation^43^. For Pt/CeO_2_-X catalysts, such absorption tail could be due to the structural disorders induced by the highly dispersed Pt species. Moreover, with the increase of the calcination temperature, the absorption intensity at 400–600 nm enhanced accordingly, suggesting that the Pt/CeO_2_ catalyst calcined at higher temperature possessed higher surface structural disorder and more surface defects, possibly resulted from the migration of Pt single atoms into CeO_2_ lattice and the stronger interaction between Pt and CeO_2_ support^44,45^. The formation of more surface defects on Pt/CeO_2_-800 was further confirmed by the in situ DRIFTS of methanol adsorption experiments (Supplementary Fig. 2), in which the band at 1034 cm^−1^ assigned to bridging methoxy species on two Ce^4+^ cations with an oxygen vacancy on Pt/CeO_2_-800 showed much higher intensity than that on pristine CeO_2_ and Pt/CeO_2_-550^46^. The indirect band gap was also calculated by Davis and Mott equation for further understanding the interaction between Pt species and CeO_2_ support within Pt/CeO_2_-X catalysts (Fig. 2d and Table 1)^43,47^. The band gap for Pt/CeO_2_-X catalysts was much lower than that for CeO_2_ support (3.23 eV), indicating the insertion of metal levels between the valence and conduction bands of CeO_2_ as well as the strong interaction through Pt–O–Ce linkage^48^. The lower band gap for Pt/CeO_2_-800 (2.61 eV) than those for Pt/CeO_2_-550 (2.96 eV) and Pt/CeO_2_-700 (2.80 eV) well supported the viewpoint that stronger Pt–O–Ce interaction was formed on Pt/CeO_2_-800 catalyst.

### Location and coordination environment of Pt single atoms

**Fig. 3:**
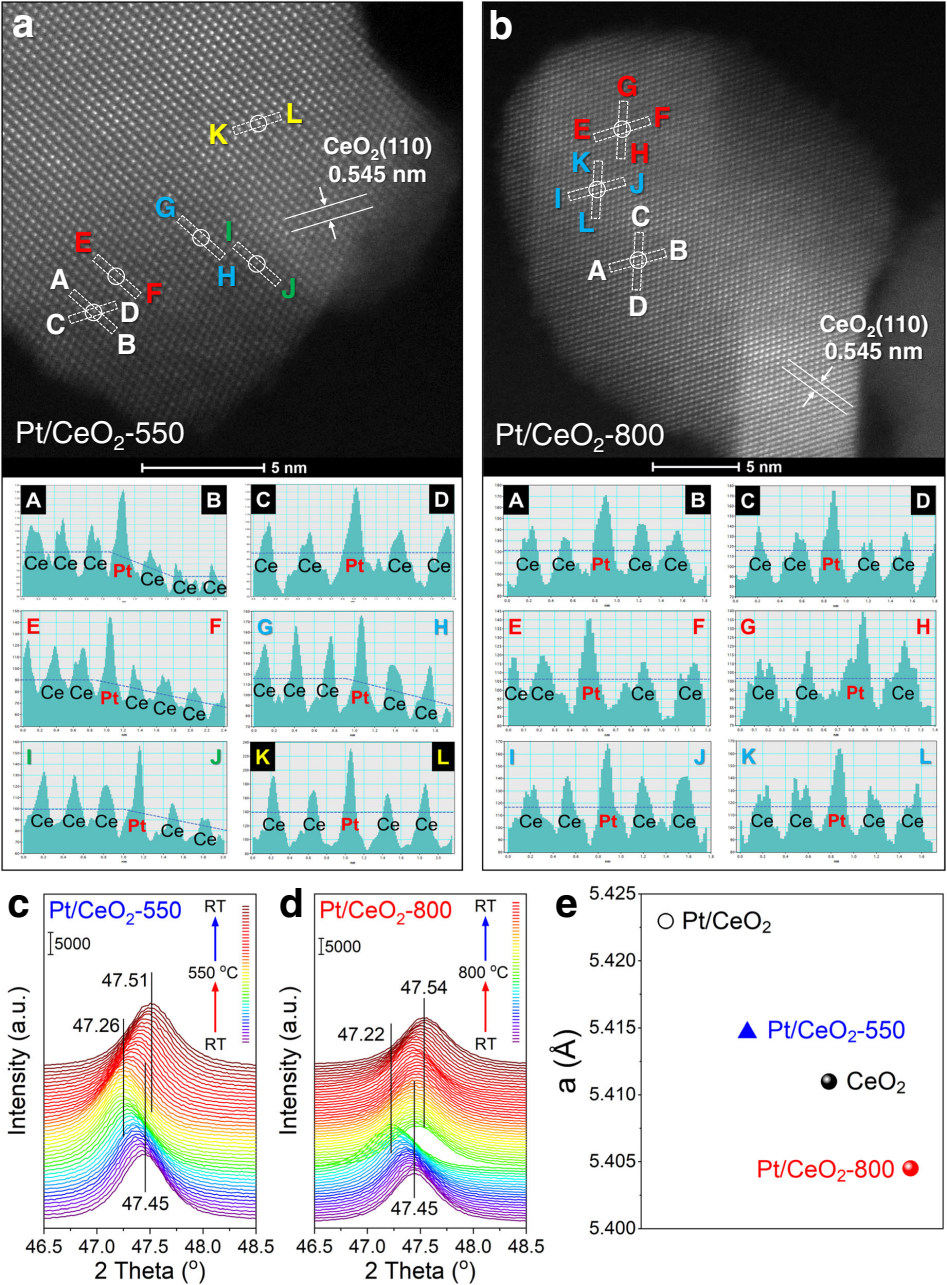
Location of Pt single atoms on CeO_2_. AC-HAADF-STEM images and the line profiles of **a** Pt/CeO_2_-550 and **b** Pt/CeO_2_-800 (additional line profiles of Pt/CeO_2_-800 can be found in Supplementary Fig. 4). In situ XRD patterns for **c** Pt/CeO_2_-550 and **d** Pt/CeO_2_-800; **e** the refined lattice parameters of Pt/CeO_2_ (without calcination), standard CeO_2_, Pt/CeO_2_-550 and Pt/CeO_2_-800 obtained by Rietveld refinements.

AC-HAADF-STEM images of Pt/CeO_2_-550 and Pt/CeO_2_-800 were collected to determine the location of Pt species (Fig. 3). CeO_2_ support was found to selectively expose (110) crystal plane. As expected, no identifiable Pt clusters were found on Pt/CeO_2_-550 and Pt/CeO_2_-800, and only bright dots assigned to isolated Pt atoms were observed. It was also shown by the high-resolution EDS mapping results that the Pt species were in highly dispersed state on both Pt/CeO_2_-550 and Pt/CeO_2_-800 (Supplementary Fig. 3). Moreover, the isolated Pt single atoms on both catalysts were found to fit well into the Ce columns on CeO_2_ (110) plane. Based on the results of in situ DRIFTS of CO adsorption, CO oxidation activity evaluation, H_2_-TPR and UV–Vis spectra, it could be deduced that the Pt single atoms on Pt/CeO_2_-550 and Pt/CeO_2_-800 should be in different status including the locations and coordination environments. Considering that the AC-HAADF-STEM images were just projection drawings, the Pt single atoms on Pt/CeO_2_-550 and Pt/CeO_2_-800 could be located at different substitution sites or epitaxial growth sites of Ce on CeO_2_ support, which enabled the Pt single atoms to be in different status but all observed in the Ce columns on CeO_2_ (110) plane. With the aid of line profiles of AC-HAADF-STEM images (Fig. 3a, b and Supplementary Fig. 4), the different locations of Pt single atoms on Pt/CeO_2_-550 and Pt/CeO_2_-800 could be well discerned. For Pt/CeO_2_-550, the Pt atoms were mainly located at the edge or step sites on the surface of CeO_2_, while the Pt atoms on Pt/CeO_2_-800 were possibly located at the Ce substitution sites on the CeO_2_ terrace (e.g., embedded into the surface lattice of CeO_2_).

To further reveal the location of Pt atoms on CeO_2_ support, two sets of in situ XRD experiments were designed, in which the XRD patterns were collected continuously throughout the sample heating (to 550 or 800 °C) and cooling process (Supplementary Fig. 5 and Fig. 3c, d). Before the measurements, the CeO_2_ powder newly impregnated with Pt nitrate solution was dried in air at 120 °C for 30 min to remove excess H_2_O (denoted as Pt/CeO_2_). During the calcination process to obtain Pt/CeO_2_-550, the XRD peaks shifted to lower angles in the heating stage from RT to 550 °C, and shifted back to higher angles in the cooling stage, which should be related to the lattice expansion and lattice contraction, respectively^49^. During the calcination process to obtain Pt/CeO_2_-800, the XRD peaks also shifted to lower angles in the heating stage (< 620 °C) due to the lattice expansion of CeO_2_ as well. However, an abrupt shift of XRD peaks back to high angles was observed for Pt/CeO_2_-800 at higher temperature of *ca*. 620 °C, which should be resulted from the desorption of O_2_ to form surface Ce_2_O_3_ species at elevated temperatures^50^. The XRD peaks for bare CeO_2_ support showed a similar shift trend during the heating and cooling stages as those for Pt/CeO_2_-550 and Pt/CeO_2_-800 catalysts (Supplementary Fig. 6). After calcination at 550 and 800 °C, the XRD diffraction peaks of Pt/CeO_2_ catalysts shifted to higher angles in varying degrees. Considering that the CeO_2_ support has already been pre-stabilized by calcination at 800 °C for 12 h, the shift in the diffraction peaks of Pt/CeO_2_ should be due to the lattice expansion or contraction of CeO_2_ induced by Pt species^51^. By conducting the Rietveld refinements of XRD patterns for Pt/CeO_2_, Pt/CeO_2_-550, and Pt/CeO_2_-800, the refined lattice parameters of CeO_2_ on these samples were calculated (Fig. 3e). It was noticeable that the lattice parameter of uncalcined Pt/CeO_2_ and Pt/CeO_2_-550 was slightly higher than that of standard CeO_2_ reference, which should result from the perturbation effect of Pt deposition on CeO_2_ surface. Interestingly, when the calcination temperature increased to 800 °C, a dramatic decrease in the lattice parameter was observed, which might be related to the substitution of Ce ions by Pt ions with smaller ionic radius (Ce^4+^ = 0.970 Å, Ce^3+^ = 1.280 Å, Pt^4+^ = 0.625 Å, Pt^2+^ = 0.800 Å) and further distortion^43,52^, in line with the previous report that the Pt^2+^ might diffuse into CeO_2_ lattice during the high temperature calcination process^43^. The in situ XRD results further suggested that the Pt atoms might have been incorporated into the surface lattice on CeO_2_ terrace within Pt/CeO_2_-800 catalyst.

**Fig. 4:**
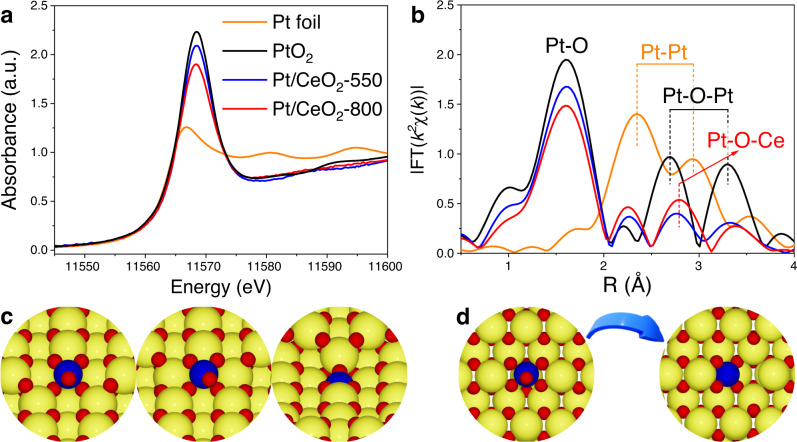
Coordination environment of Pt single atoms determined by XAS analysis. **a** Normalized XANES and **b** EXAFS magnitude of the Fourier transformed *k*^2^-weighted χ(*k*) data for Pt/CeO_2_-550 (blue) and Pt/CeO_2_-800 (red) at the Pt-L_3_ edge. Pt foil (orange) and PtO_2_ (black) references were used for XANES linear combination fitting and EXAFS comparison. **c** Three possible geometries of Pt single atoms at the edge or step sites on CeO_2_ (110) within Pt/CeO_2_-550. **d** The reconstruction of Pt single atoms on CeO_2_ (110) terrace from six-coordination sites to square-planar like coordination sites within Pt/CeO_2_-800.

X-ray absorption spectroscopy (XAS) analysis was conducted to further elucidate the valence states and the coordination environments of Pt single atoms on Pt/CeO_2_-X catalysts. As shown in Fig. 4a and Supplementary Fig. 7a, the white line (the intense absorption in the near-edge region) intensities of Pt-L_3_ XANES for the Pt/CeO_2_ catalysts were always much higher than that for Pt foil but lower than that for PtO_2_, indicating the intermediate valence state of Pt species between 0 and +4 on Pt/CeO_2_-X catalysts. Interestingly, the white line intensity of Pt-L_3_ XANES on Pt/CeO_2_ catalysts decreased monotonically as the calcination temperature increased, indicating the decreased Pt valence state accordingly. The XANES linear combination fitting was performed to further determine the average valence state of Pt species (Supplementary Fig. 8, Table 1 and Supplementary Table 1). As expected, the Pt species on Pt/CeO_2_-800 indeed showed much lower valence state (2.7 ± 0.1) than those on Pt/CeO_2_-550 (3.4 ± 0.1), which was further supported by the Pt 4f XPS results that more Pt^2+^ species were created on Pt/CeO_2_-800 comparing to those on Pt/CeO_2_-550 (Supplementary Table 2 and Supplementary Fig. 9).

Herein, to further reveal the local coordination structure of Pt single atoms on Pt/CeO_2_-550 and Pt/CeO_2_-800 catalysts, the EXAFS curve fitting was conducted and the results are plotted in both R space and *k* space (Fig. 4b, Supplementary Fig. 7b, Supplementary Fig. 10 and Supplementary Table 3). The absence of Pt–O–Pt and Pt–Pt coordination shells and the exclusive observation of Pt–O and Pt–O–Ce coordination shells further evidenced the formation of Pt single atoms on Pt/CeO_2_-550 and Pt/CeO_2_-800. Although Pt/CeO_2_-800 indeed showed a lower coordination number (CN) of Pt–O (CN_Pt–__O_ = 4.4 ± 0.3) than Pt/CeO_2_-550 (CN_Pt–__O_ = 5.1 ± 0.4), a higher CN of Pt–O–Ce was observed on Pt/CeO_2_-800 (CN_Pt–__O–__Ce_ = 4.2 ± 0.3) comparing to that on Pt/CeO_2_-550 (CN_Pt–__O–__Ce_ = 3.7 ± 0.3), further validating the formation of more Pt–O–Ce linkages within Pt/CeO_2_-800, which could be further proved by Raman spectra (Supplementary Fig. 11). As discussed above, for Pt/CeO_2_-550, the Pt single atoms should be mainly located at the edge or step sites of CeO_2_ (110), which did not induce any lattice shrinkage of CeO_2_ (Fig. 4c). In contrast, for Pt/CeO_2_-800, the significant lattice shrinkage of CeO_2_ support could be related to the incorporation of Pt atoms into the surface lattice of CeO_2_ (110). For Pt atoms substituting the surface Ce sites on CeO_2_ (110) in an ideal model, the CN_Pt–__O_ should be 6, which was higher than the CN_Pt–__O_ on Pt/CeO_2_-800 (CN_Pt–__O_ = 4.4 ± 0.3). However, since Pt^2+^ species possessed smaller ionic radius (*ca*. 0.800 Å) than Ce^4+^ (*ca*. 0.970 Å), a significant reconstruction of Pt single atoms from six-coordination sites to square-planar like coordination sites could occur on Pt/CeO_2_-800 to balance the space and charge, thus leading to the decrease in the CN_Pt–__O_ from 6 to 4 (Fig. 4d and Supplementary Fig. 12). Similar reconstruction of Pt single atoms was also reported to occur on CeO_2_ (111)^26^.

### DFT calculations on the coordination environment of Pt_1_

**Fig. 5:**
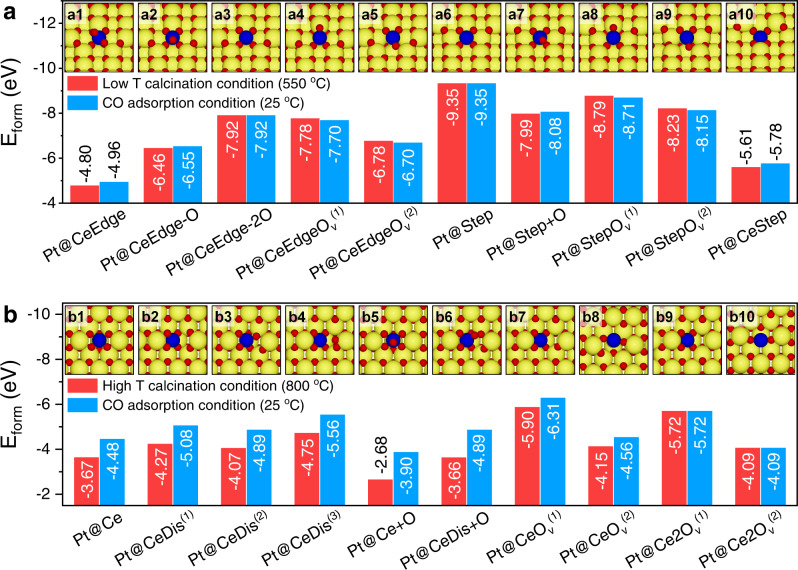
Determination of Pt_1_ configurations on CeO_2_ through DFT calculations. The formation energy (*E*_form_) of the considered Pt_1_ configurations on CeO_2_ (110) under different conditions, i.e., low-temperature (Low T) calcination condition (550 °C, 0.21 atm O_2_ pressure), high-temperature (High T) calcination condition (800 °C, 0.21 atm O_2_ pressure), and CO adsorption condition (25 °C, 10^−20^ atm O_2_ pressure) within **a** Pt/CeO_2_-550 and **b** Pt/CeO_2_-800. The insert figures are the top view of considered configurations of isolated Pt atoms at or near the edge/step sites on CeO_2_ (110) surface within Pt/CeO_2_-550 (**a1**−**10**), and at the Ce substitution sites of CeO_2_ (110) surface within Pt/CeO_2_-800 (**b1**−**10**). Large yellow, medium blue, and small red balls represented Ce, Pt, and O atoms.

To confirm the conjecture about the location, as well as the coordination environment of Pt single atoms on Pt/CeO_2_-550 and Pt/CeO_2_-800, DFT calculations were performed. For Pt/CeO_2_-550, based on the experimental evidence showing that Pt single atoms were mainly located at or near the edge/step sites on CeO_2_ (110) surface, ten configurations were proposed (Fig. 5a1–a10 and Supplementary Fig. 13). Among them, five configurations in which one Ce atom at the edge sites was replaced by one Pt atom were constructed (Fig. 5a1–a5). In Pt@CeEdge configuration, Pt replaced Ce atom at the edge sites and coordinated with 6 oxygen atoms to form octahedral Pt–O_6_ (Fig. 5a1 and Supplementary Fig. 13a). Once one or two O atoms on Pt@CeEdge were removed, the Pt@CeEdge configuration evolved into Pt@CeEdge-O or Pt@CeEdge-2O (Fig. 5a2–a3 and Supplementary Fig. 13b, c), in which Pt coordinated with 5 or 4 oxygen atoms to form Pt–O_5_ or square planar Pt–O_4_, respectively. Further removal of another oxygen atom led to the formation of Pt@CeEdgeO_v_^(1)^ (Fig. 5a4 and Supplementary Fig. 13d) or Pt@CeEdgeO_v_^(2)^ (Fig. 5a5 and Supplementary Fig. 13e) with a Pt–O_3_ coordination. Furthermore, another five configurations where Pt atoms located at or near the step sites were constructed. In the configuration of Pt@Step, Pt atom was adsorbed at the step on CeO_2_ (110) surface with a Pt–O_4_ coordination (Fig. 5a6 and Supplementary Fig. 13f). Similar to Pt@CeEdge configuration, as shown in Fig. 5a7–a9 and Supplementary Fig. 13g–i, after adding or removing O atoms, three new configurations were obtained (Pt@Step+O, Pt@StepO_v_^(1)^ and Pt@StepO_v_^(2)^). One configuration in which Pt replaced Ce step atom (Pt–O_4_ coordination) was also proposed (Fig. 5a10 and Supplementary Fig. 13j).

To compare the relative stability of the Pt structures, the formation energy of the above-mentioned configurations was calculated under two different conditions, i.e., calcination condition (550 °C, 0.21 atm O_2_ pressure) and condition at which the CO adsorption experiments were performed (25 °C, 10^−20^ atm O_2_ pressure). As illustrated in Fig. 5a, the formation energies of each configuration under the two conditions were similar, suggesting that the Pt_1_/CeO_2_ structures were almost the same after calcination and during CO adsorption process. Furthermore, other than Pt@CeEdge and Pt@CeStep, the rest eight configurations showed low formation energy, suggesting that the isolated Pt atoms could be stably anchored at edge or step sites on CeO_2_ (110) with different possible O-coordination environments.

For Pt/CeO_2_-800, based on the characterization results indicating that Pt atoms substituted the surface Ce sites on CeO_2_ (110), ten configurations of Pt_1_ on CeO_2_ (110) terrace were proposed (Fig. 5b and Supplementary Fig. 14). The most trivial configuration was the one in which Pt substituted Ce on the CeO_2_ (110) surface without any change in the arrangement of O atoms (Fig. 5b1 and Supplementary Fig. 14a), forming an asymmetrical Pt–O_5_-like coordination (Pt@Ce). However, the Pt–O_5_ structure could transform to a distorted configuration of Pt@CeDis^(1)^ without enforcing symmetry during the ionic relaxation process (Fig. 5b2 and Supplementary Fig. 14b), thus forming a Pt–O_4_ planar type of coordination. Two other distorted configurations (Pt@CeDis^(2)^ and Pt@CeDis^(3)^) were also considered. Pt@CeDis^(2)^ was similar to Pt@CeDis^(1)^ with an O atom displaced towards the top of Pt to form Pt–O_5_ configuration (Fig. 5b3 and Supplementary Fig. 14c). In Pt@CeDis^(3)^ configuration, an O atom was displaced towards a nearby O atom to form O_2_ species (Fig. 5b4 and Supplementary Fig. 14d), yielding a configuration similar to the one suggested by Tang et al.^25^. By adding an additional O atom on top of Pt in Pt@Ce and Pt@CeDis, two configurations of Pt@Ce+O (Fig. 5b5 and Supplementary Fig. 14e) and Pt@CeDis+O (Fig. 5b6 and Supplementary Fig. 14f) were achieved, with the formation of Pt–O_6_ coordination and Pt–O_5_ coordination, respectively. When one O atom was removed from Pt@CeDis, two configurations with O vacancies nearby Pt atoms were constructed, referred as Pt@CeO_v_^(1)^ (Fig. 5b7 and Supplementary Fig. 14g) and Pt@CeO_v_^(2)^ (Fig. 5b8 and Supplementary Fig. 14h), thus forming Pt–O_4_ coordination and Pt–O_3_ coordination, respectively. The further removal of one O atom from Pt@CeO_v_^(1)^ and Pt@CeO_v_^(2)^ would result in the formation of configurations with two O vacancies, which were denoted as Pt@Ce2O_v_^(1)^ (Fig. 5b9 and Supplementary Fig. 14i) and Pt@Ce2O_v_^(2)^ (Fig. 5b10 and Supplementary Fig. 14j), respectively.

The formation energy of the above-mentioned configurations was also calculated under both calcination condition and CO adsorption condition (Fig. 5b). Pt@CeDis^(1)^, Pt@CeDis^(3)^, Pt@CeO_v_^(1)^ and Pt@Ce2O_v_^(1)^ showed the lowest formation energy among the ten configurations. Furthermore, the Pt atoms in these four configurations were in Pt–O_4_ coordination. According to the results of H_2_-TPR, Raman spectra, and XAS analysis, the CN_Pt–__O_ on Pt/CeO_2_-550 was higher than that on Pt/CeO_2_-800 (5.1 vs. 4.4). Thus, the Pt_1_ species on Pt/CeO_2_-550 should be in Pt–O_4+*x*_ (*x* > 0) coordination, and the dominant Pt_1_/CeO_2_ configuration within Pt/CeO_2_-550 could be Pt@CeEdge-O or Pt@Step+O with CN_Pt–__O_ of 5. It is noteworthy that, because of the large difference in chemical potential of oxygen (−0.41 eV) between High T calcination condition and CO oxidation condition, there was noticeable difference in the formation energy of Pt_1_ under the two conditions, except for the cases with two oxygen vacancies (Fig. 5b9–b10) in which the Pt_1_ formation energy was independent of oxygen chemical potential. In contrast, there was virtually no difference in the chemical potential of oxygen (−0.08 eV) between Low T calcination condition and CO oxidation condition, resulting in the virtually identical Pt_1_ formation energy under the two conditions (Fig. 5a).

To confirm the Pt_1_/CeO_2_ configurations within Pt/CeO_2_-550 and Pt/CeO_2_-800, CO adsorption calculations were conducted. As shown in Supplementary Fig. 15a, for Pt@CeEdge-O within Pt/CeO_2_-550, CO picked up an O atom to form CO_2_ during structural (ionic) relaxation, thus forming the configuration of Pt@CeEdge-2O. CO adsorbed on Pt@CeEdge-2O showed a stretching frequency of 2047 cm^−1^, with a binding energy (*E*_B_) of −0.36 eV. When CO picked up another O atom, CO-Pt@CeEdgeO_v_^(1)^ (*E*_B_ = −1.99 eV) or CO-Pt@CeEdgeO_v_^(2)^ (*E*_B_ = −2.17 eV) was formed, which showed stretching frequencies of 2006 and 1949 cm^−1^, respectively (Supplementary Fig. 15b, c). Similar to the case of Pt@CeEdge-O, CO adsorption would pick up an O atom on Pt@Step+O to form Pt@Step (Supplementary Fig. 15d). CO-Pt@Step showed a stretching frequency of 2012 cm^−1^ (*E*_B_ = −0.38 eV). Further removal of another O atom on CO-Pt@Step resulted in the formation of CO-Pt@StepO_v_^(1)^ (*E*_B_ = −1.99 eV) and CO-Pt@StepO_v_^(2)^ (*E*_B_ = −2.34 eV), showing stretching frequencies of 2012 and 1921 cm^−1^, respectively (Supplementary Fig. 15e, f).

For Pt/CeO_2_-800, it was found that CO could not bind on the Pt atom in Pt@CeDis^(1)^, but could bind on the Ce atom adjacent to Pt atom (*E*_B_ = −0.39 eV), with a CO stretching frequency of 2102 cm^−1^ (Supplementary Fig. 16a). As reported by Tang et al.^25^, the CO adsorbed on Pt@CeDis^(3)^ structure could react with an O atom nearby, thus creating Pt@CeO_v_^(1)^ configuration. Interestingly, it was found in this study that the adsorbed CO could pull the Pt atom from the Pt–O_4_ planar configuration in Pt@CeO_v_^(1)^ to form CO–Pt–O_3_ (Supplementary Fig. 16b, *E*_B_ = −1.33 eV). The reconstruction of Pt@CeDis^(3)^ under CO adsorption condition was demonstrated in Supplementary Fig. 17. Moreover, the adsorption of CO on Pt@Ce2O_v_^(1)^ was found rather weak (*E*_B_ = −0.07 eV). Therefore, Pt@CeDis^(3)^ and Pt@CeO_v_^(1)^ were the most possible Pt_1_/CeO_2_ configurations within Pt/CeO_2_-800. Considering the lower formation energy of Pt@CeO_v_^(1)^ as compared to that of Pt@CeDis^(3)^ (Fig. 5b), Pt@CeO_v_^(1)^ should be the dominant configuration within Pt/CeO_2_-800. The formation of Pt@CeO_v_^(1)^ configuration within Pt/CeO_2_-800 was also well supported by the UV–Vis spectra that Pt/CeO_2_-800 possessed higher surface structural disorder (Fig. 2c).

Based on the in situ DRIFTS results of CO adsorption showing that the CO stretching frequency on Pt/CeO_2_-550 was higher than that on Pt/CeO_2_-800, it can be concluded that Pt@CeEdge-O was the dominant configuration within Pt/CeO_2_-550, on which the adsorbed CO showed a higher stretching frequency (2047 cm^−1^) than CO-Pt@CeO_v_^(1)^ within Pt/CeO_2_-800 (2022 cm^−1^). Moreover, the calculated E_B_ of CO-Pt@CeO_v_^(1)^ (−1.33 eV) was higher than that of CO-Pt@CeEdge-2O (−0.36 eV), which was well supported by the CO-TPD results showing that the CO adsorption on Pt/CeO_2_-800 catalyst was indeed stronger than that on Pt/CeO_2_-550 catalyst (Supplementary Fig. 18). The Bader charge analysis was conducted on Pt@CeEdge-O and Pt@CeO_v_^(1)^ configurations to further evaluate the electronic structure of Pt_1_ sites. As shown in Supplementary Fig. 19, the Pt atom in Pt@CeEdge-O representing Pt/CeO_2_-550 catalyst showed a more positive Bader charge (+1.55) than that in Pt@CeO_v_^(1)^ (+0.75) representing Pt/CeO_2_-800 catalyst, which could be resulted from the higher CN_Pt–__O_ of Pt atom in Pt@CeEdge-O. Such results were in consistence with XPS and XANES analysis suggesting that the Pt single atoms in Pt/CeO_2_-550 catalyst were in higher valence state than those in Pt/CeO_2_-800 catalyst.

### CO oxidation mechanism on Pt/CeO_2_-550 and Pt/CeO_2_-800

**Fig. 6:**
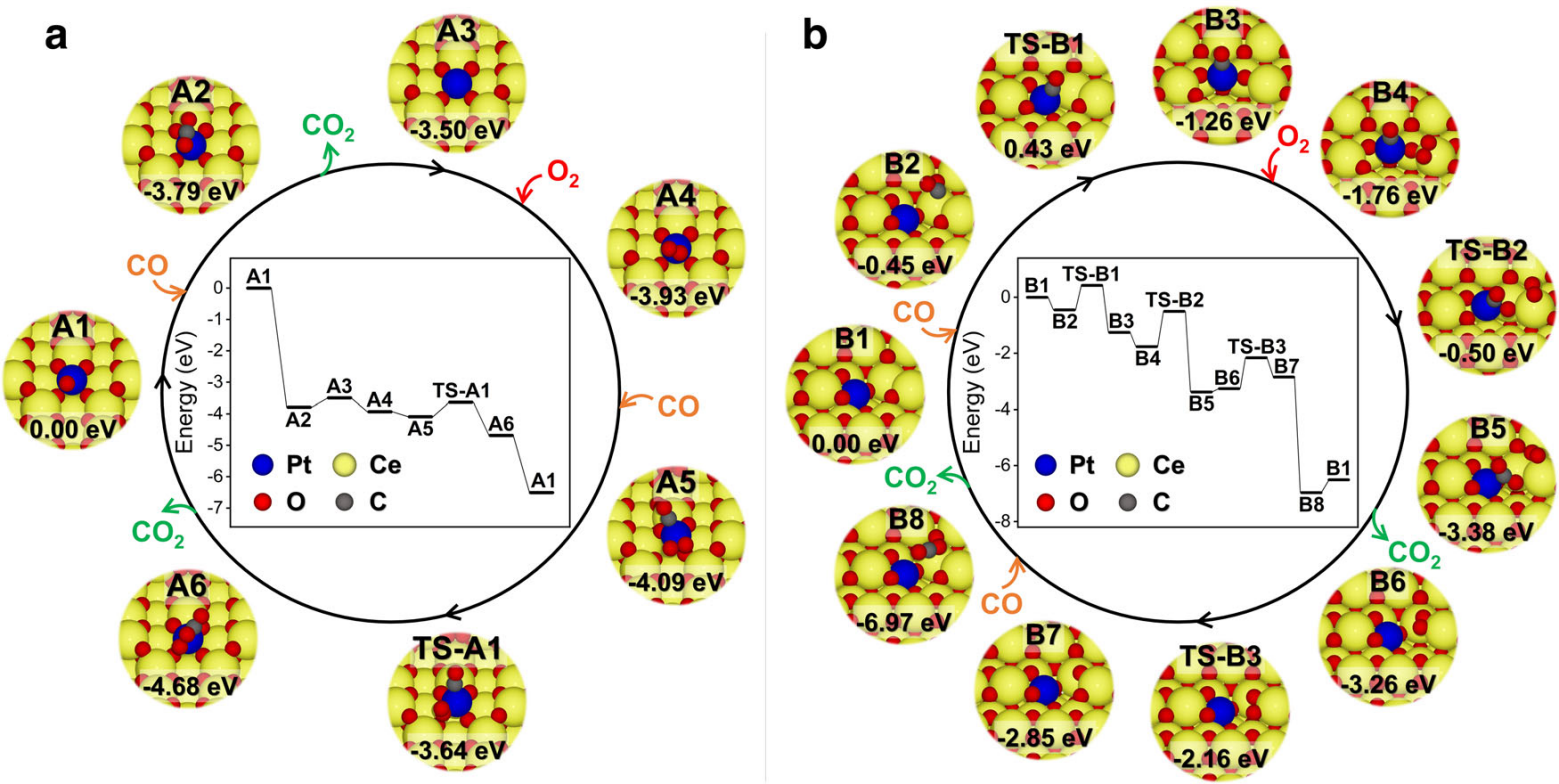
CO oxidation mechanisms on different Pt_1_/CeO_2_ catalysts. Proposed mechanisms of CO oxidation on **a** Pt@CeEdge-O (representing Pt/CeO_2_-550 catalyst) and **b** Pt@CeO_v_^(1)^ (representing Pt/CeO_2_-800 catalyst). “TS” indicates transition states. Potential energies were calculated with respect to CO and O_2_ in gas phase.

To understand the rationale for different CO oxidation activity on Pt/CeO_2_-550 and Pt/CeO_2_-800, the CO oxidation mechanisms on Pt@CeEdge-O and Pt@CeO_v_^(1)^ were studied by DFT calculations. As shown in Fig. 6a, on Pt@CeEdge-O (the dominant configuration of Pt/CeO_2_-550), CO first picked up the O atom on top of Pt (A1) to form CO_2_ that was physisorbed on the surface (A2), with a reaction energy of −3.79 eV. The CO_2_ was found to desorb from the surface with a desorption energy of 0.29 eV to form Pt@CeEdge-2O configuration (A3), followed by the adsorption of O_2_ on Pt atom with an adsorption energy of −0.43 eV (A4). Another CO was found to co-adsorb with O_2_ on Pt atom (adsorption energy = −0.16 eV) to form A5 configuration. After a transition state of TS-A1 with an activation barrier of 0.45 eV, configuration A5 could transform into A6 (reaction energy = −0.59 eV), during which CO could pick up an O atom from the adsorbed O_2_ to form CO_2_. The CO_2_ subsequently desorbed from the surface with a desorption energy of −1.82 eV, resulting in the original Pt@CeEdge-O structure.

As shown in Fig. 6b, on Pt@CeO_v_^(1)^, CO was found to adsorb on Ce atom with an oxygen vacancy (B1) to form configuration B2, with an adsorption energy of −0.45 eV. The adsorbed CO could react with Pt to pull it up forming a CO–Pt–O_3_ coordination (B3). This process was exothermic with a reaction energy of −0.81 eV and but not spontaneous with an activation barrier of 0.88 eV (TS-B1). Then, O_2_ could adsorb on a Ce atom nearby (B4) with an adsorption energy of −0.50 eV. The adsorbed CO could react with a lattice O to form CO_2_ (B5), with a reaction energy of −1.62 eV and an activation barrier of 1.26 eV (TS-B2). After the desorption of CO_2_ (desorption energy = 0.12 eV), configuration B6 with two oxygen vacancies and adsorbed O_2_ molecule was formed. O_2_ could dissociate to fill these two oxygen vacancies (B7) after overcoming an activation barrier of 1.10 eV (TS-B3). Another CO could react with one of the dissociated O atoms to form a physiosorbed CO_2_ molecule (B8). This step was highly exothermic with a reaction energy of −4.12 eV and the CO_2_ could then desorb with a desorption energy of 0.46 eV to form original Pt@CeO_v_^(1)^ structure (B1).

Comparing the above two proposed mechanisms for CO oxidation, it can be concluded that the oxidation of CO required three steps with sizable activation barrier on Pt@CeO_v_^(1)^ (the dominant structure on Pt/CeO_2_-800), while only one with a relatively lower barrier on Pt@CeEdge-O (the dominant structure on Pt/CeO_2_-550), well explaining why Pt/CeO_2_-800 showed markedly lower CO oxidation activity than Pt/CeO_2_-550. The results of dynamic oxygen storage capacity (OSC) test further confirmed that O_2_ could be better activated and consumed by CO on Pt/CeO_2_-550 than on Pt/CeO_2_-800 (Supplementary Fig. 20), well supporting the DFT calculation results that the CO oxidation and O_2_ dissociation (A5 → TS-A1 → A6) on Pt/CeO_2_-550 only needed to overcome much lower activation barrier comparing to those on Pt/CeO_2_-800 (B4 → TS-B2 → B5 and B6 → TS-B3 → B7). The lower apparent activation energy (*E*_a_) for CO oxidation on Pt/CeO_2_-550 (43 kJ∙mol^−1^) than that on Pt/CeO_2_-800 (52 kJ∙mol^−1^) (Supplementary Fig. 21) should also be resulted from the more efficient activation of reactants.

The stability of Pt/CeO_2_-550 and Pt/CeO_2_-800 under reaction conditions was evaluated in a two-round CO oxidation activity test and through comparison to Pt cluster and nanoparticle catalysts on CeO_2_. Almost no change in CO oxidation activity was observed on both Pt/CeO_2_-550 and Pt/CeO_2_-800 during the two rounds of tests up to 500 °C (both with constantly lower activity than Pt cluster and nanoparticle catalysts) (Supplementary Fig. 22), suggesting the high stability of Pt_1_ catalysts under CO oxidation atmosphere in this study. Considering the inevitable presence of water vapor in vehicle exhaust, the CO oxidation on both catalysts was also performed under wet condition. As shown in Supplementary Fig. 23, Pt/CeO_2_-550 still exhibited much higher CO oxidation activity than Pt/CeO_2_-800 under wet condition.

### NH_3_ oxidation behavior on Pt/CeO_2_-550 and Pt/CeO_2_-800

**Fig. 7:**
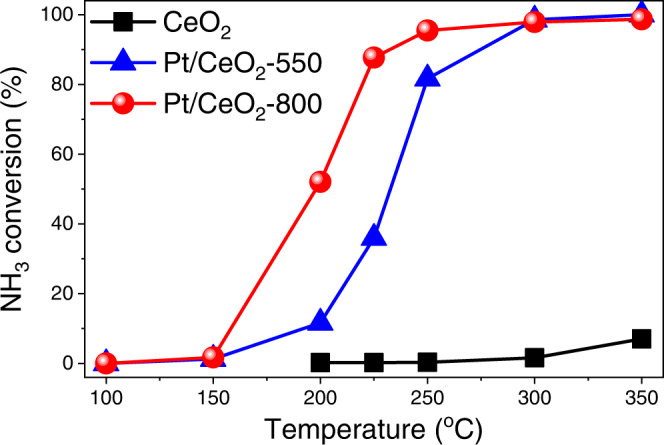
NH_3_ oxidation activity. NH_3_ oxidation performance on Pt/CeO_2_-550 and Pt/CeO_2_-800 catalysts as well as CeO_2_ support (Reaction condition: [NH_3_] = 500 ppm, [O_2_] = 5%, balanced with Ar, WHSV = 200,000 mL∙g_cat_^−1^∙h^−1^).

As discussed above, through simply controlling the calcination temperatures, Pt single atoms with different coordination environments were successfully constructed on CeO_2_. It has been demonstrated that Pt single atoms with a CN_Pt–__O_ of *ca*. 5 on Pt/CeO_2_-550 could better catalyze CO oxidation than those on Pt/CeO_2_-800 with a CN_Pt–__O_ of *ca*. 4. To further evaluate the catalytic performance of Pt/CeO_2_-550 and Pt/CeO_2_-800 in other oxidation reactions, NH_3_ oxidation, a relatively less studied but important catalytic oxidation reaction in emission control field^53,54^, was selected as a probe reaction. As shown in Fig. 7, contrary to the trend observed in CO oxidation reaction, surprisingly, Pt/CeO_2_-800 exhibited much superior NH_3_ oxidation performance than Pt/CeO_2_-550. The NH_3_ oxidation reaction on Pt/CeO_2_-800 even showed slightly higher N_2_ selectivity than that on Pt/CeO_2_-550 especially at 350 °C (Supplementary Fig. 24), and such discrepancy was mainly due to the reduced formation of non-selective oxidation products including N_2_O, NO, NO_2_ at high temperatures on Pt/CeO_2_-800 (Supplementary Fig. 25). The NH_3_ oxidation activity on CeO_2_, Pt/CeO_2_-550 and Pt/CeO_2_-800 was also tested in the presence of H_2_O. After adding 5% H_2_O to the feed gas, Pt/CeO_2_-800 still exhibited better NH_3_ oxidation activity than Pt/CeO_2_-550 (Supplementary Fig. 26).

**Fig. 8:**
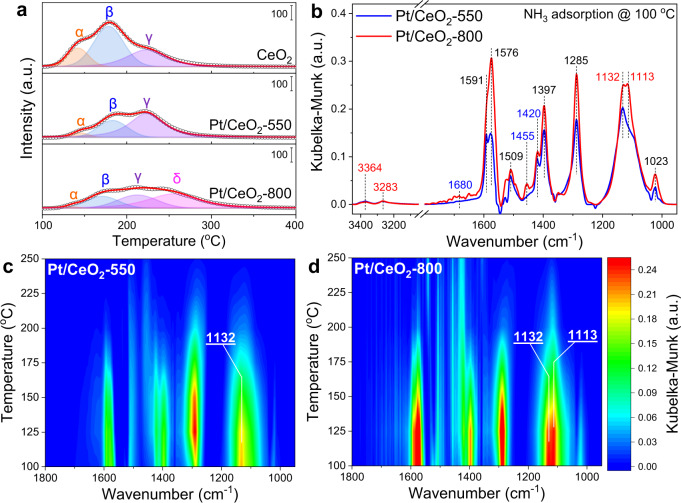
NH_3_ adsorption properties of Pt_1_/CeO_2_ catalysts. **a** NH_3_-TPD profiles on CeO_2_, Pt/CeO_2_-550, and Pt/CeO_2_-800; **b** in situ DRIFTS of NH_3_ adsorption at 100 °C; in situ DRIFTS of NH_3_ adsorption and desorption on **c** Pt/CeO_2_-550 and **d** Pt/CeO_2_-800.

To reveal the intrinsic reasons for the higher NH_3_ oxidation activity on Pt/CeO_2_-800, NH_3_-TPD was first conducted to investigate the NH_3_ adsorption capacity on pure CeO_2_ support and Pt/CeO_2_ catalysts (Fig. 8a). Three NH_3_-desorption peaks at *ca*. 130, 170 and 220 °C were observed on Pt/CeO_2_-550 (denoted as peaks α, β, and γ) similar to those observed on CeO_2_ support, which could be assigned to the desorption of NH_3_ species adsorbed on CeO_2_ sites and Pt sites with weak (α and β) or strong (γ) adsorption strength^55^. Interestingly, the peak γ on Pt/CeO_2_-550 showed a much higher relative intensity than that on CeO_2_ support, indicating that the interaction between Pt single atoms and CeO_2_ support could generate extra sites for stronger NH_3_ adsorption. More importantly, on Pt/CeO_2_-800, a new intensive NH_3_-desorption peak (δ) was clearly observed, which could be attributed to NH_3_ species more strongly bound to Pt single atoms or Ce atoms adjacent to Pt atoms on Pt/CeO_2_-800 with another unique coordination environment (i.e., Pt atoms substituting Ce sites at CeO_2_ (110) terrace). Due to the limited NH_3_ conversion below 150 °C on Pt/CeO_2_-550 and Pt/CeO_2_-800, the NH_3_ oxidation activity on both catalysts should be mainly related to the NH_3_ species strongly adsorbed on Pt sites (peaks γ and δ). As listed in Supplementary Table 4, from the deconvolution results of NH_3_-TPD profiles, a much higher ratio of NH_3_ species more strongly adsorbed on Pt sites was observed on Pt/CeO_2_-800. Given the near zero order of NH_3_ in NH_3_ oxidation reaction (Supplementary Fig. 27), the adsorption capacity of NH_3_ at the investigated temperature regime would strongly determine the NH_3_ oxidation activity on Pt/CeO_2_-550 and Pt/CeO_2_-800 catalysts. More strongly adsorbed NH_3_ species on Pt/CeO_2_-800 catalyst could be one of the main reasons for its much higher catalytic performance in NH_3_ oxidation reaction.

To further investigate the surface acidity of Pt/CeO_2_-550 and Pt/CeO_2_-800, in situ DRIFTS of NH_3_ adsorption at 100 °C was conducted (Fig. 8b). The bands at 1113, 1132, 3283 and 3364 cm^−1^ could be assigned to NH_3_ adsorbed on Lewis acid sites. The bands at 1420 and 1680 cm^−1^ could be attributed to NH_4_^+^ bound to Brønsted acid sites^55,56^. The other bands at 1023, 1285, 1397, 1509, 1576, and 1591 cm^−1^ could be assigned to nitrate species generated by the oxidation of adsorbed NH_3_^57,58^. Considering the same initial CeO_2_ support and Pt loading of Pt/CeO_2_-550 and Pt/CeO_2_-800 catalysts, the more intensive bands of nitrates on Pt/CeO_2_-800 suggested that the adsorbed NH_3_ on this catalyst could be better activated and oxidized mainly due to its unique local surface microstructure. In situ DRIFTS of NH_3_ adsorption–desorption was also conducted to further evaluate the strength of surface acid sites on Pt/CeO_2_-550 and Pt/CeO_2_-800 (Fig. 8c, d). It was clearly demonstrated that the NH_3_ bands located at 1113–1132 cm^−1^ on Pt/CeO_2_-800 were much more intensive than those on Pt/CeO_2_-550 and also showed much higher stability in Ar flow during the temperature elevation. The formation of Pt_1_ sites with different configurations on Pt/CeO_2_-550 and Pt/CeO_2_-800 should account for the distinct strength of NH_3_ adsorption on these two catalysts. Based on DFT calculations, higher adsorption energy of NH_3_ on Pt@CeO_v_^(1)^ (*E*_B_ = −1.10 eV) was observed than on Pt@CeEdge-O (*E*_B_ = −0.73 eV) (Supplementary Fig. 28), further confirming this viewpoint. Besides, it was found that NH_3_ could be preferentially adsorbed on Ce atoms adjacent to Pt atoms on both structures rather than on Pt single atoms directly.

To evaluate the reactivity of adsorbed NH_3_ on Pt/CeO_2_-550 and Pt/CeO_2_-800 catalysts, in situ DRIFTS of O_2_ reacting with pre-adsorbed NH_3_ at 175 °C was performed (Supplementary Fig. 29). As expected, more NH_3_ adsorbed on Lewis acid sites (1067, 1105 and 1132 cm^−1^) were observed on Pt/CeO_2_-800 than on Pt/CeO_2_-550 at 175 °C. With the introduction of O_2_, the NH_3_ species adsorbed on Brønsted acid sites (1424/1435 and 1650 cm^−1^) showed almost no reactivity. In contrast, the NH_3_ species coordinated to Lewis acid sites could effectively react with O_2_, and such NH_3_ species on Pt/CeO_2_-800 catalyst were consumed much faster than those on Pt/CeO_2_-550 (Supplementary Fig. 30).

**Fig. 9:**
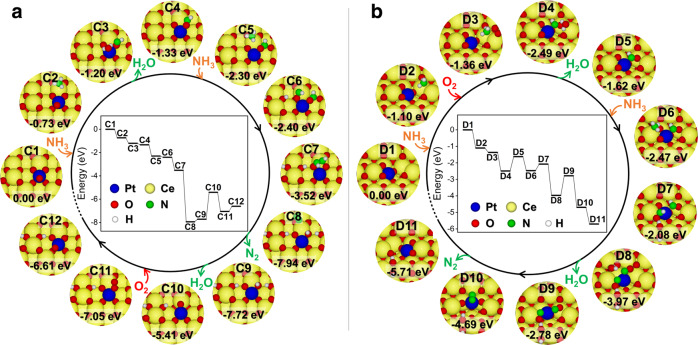
NH_3_ oxidation mechanisms on different Pt_1_/CeO_2_ catalysts. Proposed mechanisms of NH_3_ oxidation on **a** Pt@CeEdge-O (representing Pt/CeO_2_-550 catalyst) and **b** Pt@CeO_v_^(1)^ (representing Pt/CeO_2_-800 catalyst). Potential energies were calculated with respect to NH_3_ and O_2_ in gas phase.

To better reveal the intrinsic reason for the higher NH_3_ oxidation activity on Pt/CeO_2_-800, DFT calculations for mechanism study were also performed. It is worth noting that the overall stoichiometric reaction of NH_3_ oxidation is

1 $$4{{{{{{\rm{NH}}}}}}}_{3}+3{{{{{{\rm{O}}}}}}}_{2}\to 2{{{{{{\rm{N}}}}}}}_{2}+6{{{{{{\rm{H}}}}}}}_{2}{{{{{\rm{O}}}}}}$$

involving a number of steps, full consideration of which is beyond the scope of this work. Instead, we have resorted to consideration of the initial reaction

2 $$2{{{{{{\rm{NH}}}}}}}_{3}+{{{{{{\rm{O}}}}}}}_{2}\to {{{{{{\rm{N}}}}}}}_{2}+2{{{{{{\rm{H}}}}}}}_{2}{{{{{\rm{O}}}}}}+2{{{{{\rm{H}}}}}}^\ast$$

to provide some insights into the NH_3_ oxidation process. In the above reaction, there are two H atoms (H*) remaining adsorbed on the catalyst surface after N_2_ desorption. H_2_O molecules formed from the –OH groups can subsequently desorb and a surface oxygen vacancy will be created, which can be healed by the adsorption and dissociation of O_2_. More details of mechanisms and their energetics can be found in Supplementary Figs. 31 and 32, and the simplified pictures presented in Fig. 9 capture the gist of our argument.

On Pt@CeEdge-O (Fig. 9a), the NH_3_ oxidation started with the adsorption of NH_3_ on the Ce atom adjacent to Pt atom, on the upper terrace (C2). After dissociation of two N–H bonds, the resulting H atoms combined with O atom on top of Pt to form H_2_O (C3), which then desorbed from the surface leaving –NHO group behind (C4). Afterwards, the adsorption of the second NH_3_ molecule on the same Ce atom could occur (C5). After the dissociation of two N–H bonds of the second NH_3_ molecule and the diffusion of resulting H atoms, two –NHO groups were formed (C6), which could then react with each other to form N_2_H_2_ adsorbed on the surface (C7). Subsequently, N_2_ could be formed after the dissociation of the two N–H bonds (C8) and then desorbed from the surface leaving four H atoms on the catalyst (C9). The followed step was the formation and desorption of second H_2_O molecule, thus creating an oxygen vacancy (C10). The vacancy could be filled by the adsorption of O_2_ (C11). After reconstruction, the original structure with the addition of two H atoms on the upper terrace was achieved (C12).

A similar process was observed on Pt@CeO_v_^(1)^ as well (Fig. 9b). The oxidation of NH_3_ on Pt@CeO_v_^(1)^ started with the adsorption of NH_3_ on the Ce atom adjacent to Pt atom with an oxygen vacancy (D2), followed by the co-adsorption of O_2_ to form D3 configuration. After the dissociation of two N–H bonds and O_2_ molecule, a H_2_O molecule was formed (D4), which could desorb from surface leaving –NHO group behind (D5). Then, the second NH_3_ molecule could adsorb on another Ce atom nearby the Pt atom (D6). After the dissociation of two N–H bonds of the second NH_3_ molecule, two –NHO groups were formed on Pt@CeO_v_^(1)^ (D7). The two H atoms from these two –NHO groups could recombine with surface O atom to form second H_2_O (D8), which could desorb from surface leaving two –NO groups and an oxygen vacancy (D9). The formed N_2_ from these two –NO groups could then desorb from surface (D10). After a surface structural reconstruction, the catalyst could return to its starting configuration with two additional H atoms on the terrace of the CeO_2_ (110) surface (D11).

When comparing the two reaction mechanisms mentioned-above, it can be observed that the N atoms in –NHO or –NO groups on Pt@CeO_v_^(1)^ (representing Pt/CeO_2_-800 catalyst) were coordinated to Pt atom, which could weaken the strength of Pt–O bond and accordingly facilitate the formation (D7 → D8) and desorption (D8 → D9) of the second H_2_O molecule. The desorption energy of the second H_2_O formed on Pt@CeO_v_^(1)^ was determined as 1.19 eV, much lower than that on Pt@CeEdge-O representing Pt/CeO_2_-550 catalyst (2.31 eV) on which the N atoms in –NHO or –NO groups were coordinated to Ce atoms. For the NH_3_ oxidation on both Pt@CeEdge-O and Pt@CeO_v_^(1)^, the desorption of the second H_2_O molecule (C9 → C10 and D8 → D9, respectively) was the rate limiting step. Therefore, the lower desorption energy of the second H_2_O molecule on Pt@CeO_v_^(1)^ was one of the main reasons for the much higher NH_3_ oxidation activity obtained on Pt/CeO_2_-800.

## Discussion

Pt single atom catalysts with different local coordination environments on CeO_2_ support were finely fabricated by a simple calcination temperature control strategy. With the calcination temperature increased from 350 to 800 °C, the Pt clusters could first disperse into Pt single atoms, and then the Pt single atoms with much stronger interaction with CeO_2_ through Pt–O–Ce linkages were created. Different from Pt/CeO_2_-550 catalyst in which the Pt single atoms were mainly located at the edge sites of CeO_2_ support, for Pt/CeO_2_-800 catalyst, the Pt single atoms were mainly incorporated into the surface lattice of CeO_2_ with further structural reconstruction to form a square-planar like coordination environment on CeO_2_ terrace. Although the Pt single atoms on Pt/CeO_2_-800 catalyst showed rather limited catalytic activity in CO oxidation reaction due to its inferior ability to activate O_2_, the enhanced surface acidity for NH_3_ adsorption/activation and the easier desorption of H_2_O produced during the reaction enabled Pt/CeO_2_-800 to exhibit much higher NH_3_ oxidation activity than that on Pt/CeO_2_-550. It is suggested that the precise control of the coordination environments of Pt single atoms is beneficial for maximizing their catalytic performance in different oxidation reactions. This work can provide instructive insights into the flexible tuning of local coordination environments of single atom catalysts for more efficient application in different target reactions.

## Methods

### Catalyst preparation

Before being used as a support, commercial CeO_2_ (high surface area ceria with the BET surface area as *ca*. 120 m^2^/g) was pre-calcined in air at 800 °C for 12 h to minimize its potential structural changes in the subsequent catalyst preparation process. To prepare CeO_2_ supported Pt catalysts, 1.00 wt.% Pt using Pt(NO_3_)_2_ as precursor was loaded onto the pretreated CeO_2_ support by incipient wetness impregnation (IWI) method. After impregnation of Pt(NO_3_)_2_ onto CeO_2_, the wet powder was dried in air at 120 °C for 1 h. Afterwards, the obtained powder was calcined in air at 350, 550, 700, or 800 °C for 2 h. The obtained catalysts were denoted as Pt/CeO_2_-X, with X °C as calcination temperature. The practical mass loadings of Pt in the selected catalysts were determined by inductively coupled plasma-optical emission spectrometry (ICP-OES). As expected, Pt/CeO_2_-550 (0.92 wt.%) and Pt/CeO_2_-800 (0.90 wt.%) showed almost the same Pt loadings, which were very close to the nominal Pt loading (1.00 wt.%).

### Characterizations

The specific surface area and pore volume were measured by N_2_ physisorption at −196 °C using a Micromeritics ASAP-2020 analyzer. Before each test, the samples were degassed at 300 °C for 4 h. The specific surface area was calculated using Brunauer-Emmett-Teller method, and the pore volume and pore size distribution were determined by BJH method using the desorption isotherms.

In situ diffuse reflectance infrared Fourier transform spectroscopy (in situ DRIFTS) study was carried out on a Thermo Nicolet iS50 FTIR spectrometer equipped with a liquid nitrogen-cooled mercury–cadmium–telluride (MCT) detector. In each test, 50 mg of catalyst powder was placed in the DRIFTS cell (PIKE Technologies DiffusIR), pressed, and mounted. The catalysts were first pretreated in air at 300 °C for 1 h. Afterwards, the catalysts were cooled down step-wise to room temperature. During this process, the background spectra were collected at target temperatures for different experiments (e.g., 25 °C for CO adsorption, 25 °C for methanol adsorption, 100/125/150/175/200/225/250 °C for NH_3_ adsorption/desorption, and 175 °C for NH_3_ oxidation). For CO adsorption, methanol adsorption, or NH_3_ adsorption/desorption/oxidation experiments, the feed gas consisted of 1% CO, 15% methanol, or 500 ppm NH_3_ and/or 5 vol% O_2_ (when used), respectively, using Ar as balance. The total flow rate was controlled at 83.33 mL∙min^−1^. All the spectra were collected from 400 to 4000 cm^−1^ at a resolution of 4 cm^−1^ for 100 scans.

H_2_-temperature-programmed reduction (H_2_-TPR) was conducted on a Quantachrome Autosorb-iQ instrument. Typically, 30 mg of sample was loaded in a U-type quartz tube reactor and pretreated with air at 400 °C for 1 h. When the sample was cooled down to room temperature in air, the feeding gas was switched to 10 vol% H_2_ balanced with Ar (30 mL∙min^−1^). Then, the reactor was heated linearly to 900 °C with a ramping rate of 10 °C∙min^−1^. The signal of H_2_ consumption was monitored by a thermal conductivity detector (TCD). H_2_O moisture in the gas mixture was removed by a cold trap filled with liquid N_2_ before passing into the TCD.

Ultraviolet-visible (UV–Vis) spectra were collected on a Shimadzu UV-2401 PC instrument in diffuse mode. BaSO_4_ was used as a reference and the spectra collection range was 200−800 nm. Samples were exposed to air at room temperature throughout the experiments.

Atomic-resolution aberration-corrected high angle annular dark field scanning transmission electron microscopic (AC-HAADF-STEM) images were collected on a FEI Titan Cubed G2 60-300 aberration-corrected S/TEM instrument at 300 kV accelerating voltage. The observations were performed in the HAADF mode, which allowed Z-contrast imaging. The probe convergence angle on the Titan electron microscope was 22 mrad, and the angular range of the HAADF detector was from 79.5 to 200 mrad. To prepare the TEM samples, an appropriate amount of sample powder was dispersed in ethanol and then dropped on a 3 mm TEM Mo grid. The images of energy dispersive spectroscopy (EDS) elemental mapping in the STEM mode were obtained from the Titan electron microscope using SuperX system.

In situ XRD experiments were conducted on a Rigaku SmartLab SE XRD instrument equipped with a high speed 1D silicon strip detector (D/teX Ultra 250, Rigaku, Japan) and an in situ cell (Reactor X, Rigaku, Japan). X-rays were generated from a Cu source, with a tube operational power of 2.2 kW (Cu Kα). Additional PXRD parameters were as follows: goniometer radius = 300 mm, incident and receiving Soller slits = 2.5 °, length limiting slit = 10 mm, with a receiving slit Ni filter mitigating the Cu Kβ signal. The temperature of the sample was controlled by IR heating of the quartz sample holder (sample indention dimensions: 13 × 20 × 0.4 mm) from below, and the gas composition in the cell was controlled by a bank of mass flow controllers (Brooks 5850e, Brooks Instrument, Hatfield, PA, USA). During the experiments, dry air flowed through the in situ cell (100 mL∙min^−1^). Samples were held on at 25 °C for 5 min, and then the XRD scans were performed continuously throughout the temperature ramping, holding and cooling steps. In more detail, the samples were heated from 25 °C to the target temperatures (550 or 800 °C) with a ramping rate of 2 °C∙min^−1^, held at the target temperatures (550 or 800 °C) for 2 h, and finally cooled to 25 °C at a rate of 2 °C∙min^−1^. The XRD scans were taken from 25 to 50° at a scan rate of 2 °C∙min^−1^, with a step size of 0.01°.

ICP-OES measurement was conducted on an Optima 8300 instrument (PerkinElmer). The incident power was set at 13,000 W and the wavelength for Pt detection was 265.945 nm.

XAS for Pt L_3_-edge in fluorescence mode was measured at beamline 7-BM QAS of the National Synchrotron Light Source II (NSLS-II) at Brookhaven National Laboratory. The energy range of the X-ray provided by this beamline was 4.7–31 keV. Its monochromator is equipped with a Si(111) channel-cut crystal and runs at continuous scan mode. The duration of a typical scan was 30 s and each sample was scanned 40 times. Samples were exposed to air at room temperature throughout the experiments. The XAS data including X-ray absorption near edge structure (XANES) and extended X-ray absorption fine structure (EXAFS) were analyzed using Demeter software package.

X-ray photoelectron spectroscopy (XPS) was collected on a VG CLAM 4 MCD analyzer. Before the analysis, the samples were degassed in a preparation chamber (10^−5^ Torr) for 0.5 h. Then, the samples were put into the analysis chamber (3 × 10^−9^ Torr) for further analysis. The binding energies (BE) were calibrated with C1s line at 284.6 eV.

Raman spectra were collected on a Renishaw Laser Raman spectrometer (Renishanplc) with an Ar^+^ laser beam. The emission line was 532 nm and the output power was 10 mW. Samples were exposed to air at room temperature throughout the experiments.

CO-temperature programmed desorption (CO-TPD) was conducted in a fixed-bed quartz tube flow reactor with an online mass spectrometer (Hiden Analytical, HPR20 R&D). The mass-to-charge ratio (*m*/*z*) of 28 was used to monitor CO. In each test, 100 mg sample was pretreated by air at 300 °C for 1 h and then saturated with CO at 30 °C. Then, Ar was introduced into the quartz tube reactor to remove the weakly adsorbed CO. Finally, the sample was heated linearly to 300 °C at a rate of 10 °C∙min^−1^ in flowing Ar.

The dynamic oxygen storage capacity (OSC) was measured by a multi-pulse experiment under alternating pulses of 2% CO/4% O_2_ with a total flow rate of 100 mL∙min^−1^. Before testing, all samples were pretreated in air at 300 °C for 1 h. A single cycle lasted 1 min, with 30 s CO/30 s O_2_ cycling switch controlled by automatic mass flow controller (MFC) systems. The OSC values were calculated from the averaged CO_2_ formation in a cycle (30 s + 30 s) detected by an online mass spectrometer (Hiden Analytical, HPR20 R&D).

NH_3_-temperature-programmed desorption (NH_3_-TPD) was carried out in a fixed-bed quartz flow reactor connected with an online Thermo Nicolet iS10 FTIR spectrometer equipped with a 2 m path-length gas cell (200 mL volume). In each test, 100 mg sample was pretreated by pure Ar at 200 °C for 1 h and then saturated with NH_3_ at 100 °C. Then, Ar was introduced into the quartz tube reactor again to remove the weakly adsorbed NH_3_. Finally, the sample was heated linearly to 500 °C at a rate of 10 °C∙min^−1^ in the flowing Ar (100 mL∙min^−1^).

### Details of density functional theory (DFT) based calculations

DFT calculations were performed using the Vienna ab initio Simulation Package (VASP)^59^, employing the projector-augmented wave (PAW)^60,61^ and plane-wave basis set. The generalized-gradient approximation (GGA) was used in the form of the Perdew-Burke-Ernzerhof (PBE)^62^ functional together with the DFT-D3 correction^63^ to describe electronic exchange-correlation. The DFT + U method with U = 5 eV for Ce 5f orbitals^64^ was used to properly describe the electronic structure of CeO_2_. The electron kinetic energy cut-off for plane-wave expansion was set to 500 eV. For modeling the CeO_2_ (110) surface, a 5 layer 4 × 3 CeO_2_ (110) slab was constructed, i.e., 12 Ce and 24 O atoms per layer, using the optimized lattice constant of 5.479 Å. For modeling the stepped CeO_2_ (110) surface, a twice-large slab was removed with half of the top CeO_2_ layer to create the step. 15 Å vacuum added along the surface normal direction was used to decouple periodical images along the surface normal direction. Given the large size of the supercell, the Brillouin zone was sampled with one point at the zone center, using a Gaussian smearing with σ = 0.1 eV for structural relaxation. All electronic cycles converged to 10^−5^ eV. All structures were relaxed to minimize the stress until all components of forces acting on each atom were less than 10^−2^ eV/Å.

To search the preferable location of Pt atom on surface, various surface Ce atoms were replaced with Pt atoms. To mimic the cases that Pt atom was next to O-vacancies, one or two O atoms nearby Pt were removed to create a scenario. To mimic the cases that Pt atom was coordinated with 5 or 6 O atoms, one O atom was added on top of Pt. For step surface, Pt atoms adsorbed at the step sites were also considered.

To evaluate the relative formation energy of Pt_1_ configurations, the formation energy of Pt_1_/CeO_2_ was defined as

3 $${E}_{{{{{\rm{form}}}}}}={F}_{{{{{{\rm{Pt}}}}}}_{1}/{{{{{\rm{CeO}}}}}}_{2}}-{F}_{{{{{{\rm{CeO}}}}}}_{2}},$$

where ${F}_{{{{{{{\rm{Pt}}}}}}}_{1}/{{{{{\rm{CeO}}}}}}_{2}}$ and ${F}_{{{{{{\rm{CeO}}}}}}_{2}}$ were the free energy of Pt_1_/CeO_2_ system and CeO_2_ surface. The free energy of a system was defined as

4 $$F=E+{F}_{{{{{\rm{Vib}}}}}}+PV-{N}_{{{{{\rm{Ce}}}}}}{{\mu }_{{{{{\rm{Ce}}}}}}}^{o}-{N}_{{{{{\rm{O}}}}}}{\mu }_{{{{{\rm{O}}}}}}-{N}_{{{{{\rm{Pt}}}}}}{\mu }_{{{{{\rm{Pt}}}}}},$$

where $E$, ${F}_{{{{{{\rm{Vib}}}}}}}$, and ${PV}$ were the total energy (calculated from DFT), the vibrational contribution to free energy, and the contribution to free energy of the change of pressure and volume, respectively. The difference between ${PV}$ term of Pt_1_/CeO_2_ and clean CeO_2_ surface was small and could be ignored. In this work, the difference in ${F}_{{{{{{\rm{Vib}}}}}}}$ between Pt_1_/CeO_2_ and clean CeO_2_ surface was not included for computational feasibility, as 20 Pt_1_/CeO_2_ configurations with large simulation supercell were considered. Nevertheless, such contribution was expected to play a minor role because of the large difference between the formation energy of considered Pt_1_/CeO_2_ configurations, as shall be seen. ${N}_{X}$ and ${\mu }_{X}$ were the number of *X* atoms in the considered system and chemical potential of *X* atom (*X =* Ce, O, or Pt), respectively. As Ce was in equilibrium with Ce atoms in bulk CeO_2_ (i.e., if Ce atoms moved away from the surface, they would go to bulk CeO_2_), ${\mu }_{{{{{{\rm{Ce}}}}}}}$ and ${\mu }_{{{{{\rm{O}}}}}}$ were not independent and

5 $${\mu }_{{{{{\rm{Ce}}}}}}+2{\mu }_{{{{{\rm{O}}}}}}={E}_{{{{{{\rm{CeO}}}}}}_{2}},$$

where ${E}_{{{{{{{\rm{CeO}}}}}}}_{2}}$ was the total energy per bulk CeO_2_ unit. As oxygen was in equilibrium with the O_2_ reservoir surrounding the surface, the chemical potential of oxygen at temperature $\it {{{{T}}}}$ and partial pressure ${p}_{{{{{{\rm{O}}}}}}_{2}}$ was calculated as

6 $${\mu }_{{{{{\rm{O}}}}}}(T,p)=1/2({E}_{{{{{\rm{Tot}}}}}}^{{{{{{\rm{O}}}}}}_{2}}+{E}_{{{{{{\rm{ZPE}}}}}}}^{{{{{{\rm{O}}}}}}_{2}})+{\tilde{\mu }}_{{{{{\rm{O}}}}}}(T,{p}^{o})+1/2{k}_{B}T\,{{{{{\mathrm{ln}}}}}}({p}_{{O}_{\rm2}}/{p}^{o}),$$

where ${E}_{{{{{\rm{Tot}}}}}}^{{{{{{\rm{O}}}}}}_{2}}$ and ${E}_{{{{{\rm{ZPE}}}}}}^{{{{{{\rm{O}}}}}}_{2}}$ were the total energy and zero point energy of an O_2_ molecule calculated with DFT, ${k}_{B}$ was Boltzmann constant, ${p}^{o}$ was a reference pressure, and ${\tilde{\mu }}_{O}(T,{p}^{o})$ was the temperature dependent part that could be calculated from experimental data^65,66^. As Pt was often deposited on the surface at the beginning of the calcination process, there was no reservoir for Pt during or after calcination, thus the chemical potential of Pt was approximated as the total energy of the isolated Pt atom.

The adsorption energy of CO on Pt_1_/CeO_2_ was calculated as

7 $${E}_{{{{{\rm{Ads}}}}}}={E}_{{{{{{\rm{CO}}}}}}-{{{{{\rm{Pt}}}}}}_{1}/{{{{{\rm{CeO}}}}}}_{2}} - {E}_{{{{{{\rm{Pt}}}}}}_{1}/{{{{{\rm{CeO}}}}}}_{2}} - {E}_{{{{{\rm{CO}}}}}},$$

where ${E}_{{{{{{\rm{CO}}}}}}-{{{{{{\rm{Pt}}}}}}}_{1}/{{{{{\rm{Ce}}}}}}{{{{{\rm{O}}}}}}_{2}}$, ${E}_{{{{{{{\rm{Pt}}}}}}}_{1}/{{{{{\rm{Ce}}}}}}{{{{{\rm{O}}}}}}_{2}}$ and ${E}_{{{{{{\rm{CO}}}}}}}$ were the total energy of CO adsorption configuration, Pt_1_/CeO_2_ system, and an isolated CO molecule which was evaluated inside a cubic 15 × 15 × 15 Å^3^ box, respectively. CO stretching frequencies were calculated using the finite-difference method as implemented in the Phonopy code (a 0.01 Å displacement was used, and electronic cycles were converged to 10^−6^ eV)^67^. It was found that displacements applied to about 30 atoms around the adsorbed CO molecule were sufficient to obtain converged CO stretching frequencies.

Since the study here involved considerations of a large number of configurations of adsorbed reactants and their intermediates as appropriate for CO oxidation and NH_3_ oxidation on Pt@CeO_v_^(1)^ and Pt@CeEdge-O, for computational feasibility, a CeO_2_ (110) slab with 4 layers of 4 × 3 CeO_2_ (110), i.e., 12 Ce and 24 O atoms per layer, was used for the investigations on Pt@CeO_v_^(1)^. A CeO_2_ (110) slab with 5 layers of 1 × 3 CeO_2_ (230) stepped surface, i.e., 12 Ce and 24 O atoms per layer, was used for CO oxidation study on Pt@CeEdge-O. A CeO_2_ (110) slab with 4 layers of 6 × 3 CeO_2_ (110) with 50% of the top layer removed, creating the step in the Pt@CeEdge-O model, was used for NH_3_ oxidation study. Transition state (TS) for a reaction was determined using the nudged-elastic band (NEB) and the climbing-image (CI-) NEB methods^68,69^.

### Catalytic activity test

The CO oxidation and NH_3_ oxidation activities of the catalysts were evaluated in a fixed-bed quartz tube reactor. Samples (40–60 mesh) were mixed with SiC to minimize the heat effect (with a mass ratio of 1:10). For CO oxidation activity test, the reactants consisted of 1% CO, 1% O_2_ and 5% H_2_O (if used) in Ar balance. The total flow rate was 83.33 mL∙min^−1^ with a weight hourly space velocity (WHSV) of 400,000 mL∙g_cat_^−1^·h^−1^. The outlet gas was detected by an online mass spectrometer (Hiden Analytical, HPR20 R&D). The mass-to-charge ratio (*m*/*z*) of 28 and 44 were used to monitor CO and CO_2_, respectively. For NH_3_ oxidation activity test, the reactants consisted of 500 ppm NH_3_, 5% O_2_, and 5% H_2_O (if used) in Ar balance. The WHSV was controlled at 200,000 mL∙g_cat_^−1^·h^−1^. The concentrations of NH_3_, NO, NO_2_, and N_2_O in the outlet gas were measured by Thermo Nicolet iS10 FTIR spectrometer equipped with a 2 m path-length gas cell with 200 mL volume. All reactions were performed under steady state conditions. The CO conversion in CO oxidation reaction, and the NH_3_ conversion and N_2_ selectivity in NH_3_ oxidation reaction were determined according to the following equations:

8 $${{{{{\rm{CO}}}}}}\,{{{{{\rm{conversion}}}}}} \, (\%)=\{({[{{{{{\rm{CO}}}}}}]}_{{{{{\rm{in}}}}}}-{[{{{{{\rm{CO}}}}}}]}_{{{{{\rm{out}}}}}})/{[{{{{{\rm{CO}}}}}}]}_{{{{{\rm{in}}}}}}\}\times 100\%$$

9 $${{{{{\rm{NH}}}}}}_{3}\,{{{{{\rm{conversion}}}}}} \, (\%)=\{({[{{{{{\rm{NH}}}}}}_{3}]}_{{{{{\rm{in}}}}}}-{[{{{{{\rm{NH}}}}}}_{3}]}_{{{{{\rm{out}}}}}})/{[{{{{{\rm{NH}}}}}}_{3}]}_{{{{{\rm{in}}}}}}\}\times 100\%$$

10 $${{{{{\rm{N}}}}}}_{2}\,{{{{{\rm{selectivity}}}}}} \, (\%)=\{1-({[{{{{{\rm{NO}}}}}}]}_{{{{{\rm{out}}}}}}+{[{{{{{\rm{NO}}}}}}_{2}]}_{{{{{\rm{out}}}}}}+2{[{{{{{\rm{N}}}}}}_{2}{{{{{\rm{O}}}}}}]}_{{{{{\rm{out}}}}}})/({[{{{{{\rm{NH}}}}}}_{3}]}_{{{{{\rm{in}}}}}}-{[{{{{{\rm{NH}}}}}}_{3}]}_{{{{{\rm{out}}}}}})\}\times 100\%$$

In the reaction kinetics study, to avoid the heat and mass transfer effects, the reaction rates were determined with CO or NH_3_ conversions below 15%, and samples (40–60 mesh) were mixed with SiC to minimize the heat effect (with a mass ratio of 1:20) before the test. For CO oxidation reaction, the feed gas consisted of 1% CO and 1% O_2_ in Ar balance. The WHSV was controlled at 800,000 mL∙g_cat_^−1^·h^−1^ and 400,000 mL∙g_cat_^−1^·h^−1^ for Pt/CeO_2_−550 and Pt/CeO_2_−800, respectively. For NH_3_ oxidation reaction, the feed gas consisted of 500 ppm NH_3_ and 5% O_2_ in Ar balance, with a WHSV of 600,000 mL∙g_cat_^−1^·h^−1^.

## Supplementary information

Supplementary Information

## Data Availability

Source data are provided with this paper.

## Source data

Source Data
