# Supplementary material for: Fine-tuned local coordination environment of Pt single atoms on ceria controls catalytic reactivity
Source: Nat Commun. 2022 Nov 18;13:7070. doi: 10.1038/s41467-022-34797-2 (PMC9674627; doi:10.1038/s41467-022-34797-2)
Supplement: Supplementary file 1 — Supplementary Information [file 41467_2022_34797_MOESM1_ESM.pdf]

Supplementary Information

Fine-tuned local coordination environment of Pt single atoms on ceria controls catalytic reactivity

Wei Tan[1,2,†], Shaohua Xie[1,†], Duy Le[3,†], Weijian Diao[4], Meiyu Wang[5], Ke-Bin Low[6], Dave Austin[3], Sampyo Hong[7], Fei Gao[2], Lin Dong[2], Lu Ma[8], Steven N. Ehrlich[8], Talat S. Rahman[3], Fudong Liu[1,*]

[1] *Department of Civil, Environmental, and Construction Engineering, Catalysis Cluster for Renewable Energy and Chemical Transformations (REACT), NanoScience Technology Center (NSTC), University of Central Florida, Orlando, FL 32816, United States*

[2] *State Key Laboratory of Pollution Control and Resource Reuse, School of the Environment; Jiangsu Key Laboratory of Vehicle Emissions Control, School of Chemistry and Chemical Engineering; Center of Modern Analysis, Nanjing University, Nanjing 210023, China*

[3] *Department of Physics, Catalysis Cluster for Renewable Energy and Chemical Transformations (REACT), University of Central Florida, Orlando, FL 32816, United States*

[4] *Department of Chemical and Biological Engineering, Villanova University, Villanova, PA 19085, United States*

[5] *College of Engineering and Applied Sciences, Nanjing University, Nanjing, 210093, China*

[6] *BASF Corporation, Iselin, NJ 08830, United States*

[7] *Brewton-Parker College, Mount Vernon, GA 30445, United States*

[8] *National Synchrotron Light Source II (NSLS-II), Brookhaven National Laboratory, Upton, NY 11973, United States*

[†] Wei Tan, Shaohua Xie and Duy Le contributed equally to this work.

*Corresponding author: fudong.liu@ucf.edu (F. Liu); Tel: 407-823-6219; Fax: 407-823-3315.

Supplementary Table 1 Linear combination fitting results of Pt L_3-edge XANES for Pt/CeO_2-X catalysts (X = 350, 550, 700 and 800).

Samples	Ratio of PtO_2	Ratio of Pt foil	Averaged valence state
**Pt/CeO_2-350**	0.906 ± 0.013	0.094 ± 0.009	3.6 ± 0.1
**Pt/CeO_2-550**	0.839 ± 0.013	0.161 ± 0.010	3.4 ± 0.1
**Pt/CeO_2-700**	0.733 ± 0.014	0.267 ± 0.011	2.9 ± 0.1
**Pt/CeO_2-800**	0.676 ± 0.013	0.324 ± 0.010	2.7 ± 0.1

Supplementary Table 2 Surface atomic concentration and surface chemical states determined by XPS.

Samples	Surface atomic concentration (%)			Ce^{3+}/total Ce (%)	Pt^{2+}/total Pt (%)
	O	Ce	Pt		
**CeO_2**	71.3	28.7	-	19.2	-
**Pt/CeO_2-350**	71.2	28.4	0.4	18.4	36.4
**Pt/CeO_2-550**	71.2	28.0	0.8	16.4	79.2
**Pt/CeO_2-700**	70.5	28.4	1.1	18.0	86.8
**Pt/CeO_2-800**	71.0	27.9	1.1	17.2	87.5

Supplementary Table 3 EXAFS fitting results for Pt/CeO_2-X catalysts using Pt foil, PtO_2 as references.

Sample	Shell	CN[a]	R (Å)[b]	ΔE_0 (eV)[c]	σ^2 (10^{-3} Å^2)[e]	R-factor[e]
**Pt foil**	Pt-Pt	12.0 ± 0.8	2.76 ± 0.01	8.0 ± 0.5	4.05 ± 0.53	0.003
	Pt-O	6.0 ± 0.7	2.01 ± 0.01	9.5 ± 1.1	1.77 ± 1.42	
**PtO_2**	Pt-O-Pt	6.0 ± 0.7	3.10 ± 0.01	9.5 ± 1.1	1.39 ± 1.37	0.010
	Pt-O	12.0 ± 1.4	3.65 ± 0.01	9.5 ± 1.1	7.88 ± 5.21	
**Pt/CeO_2-550**	Pt-O	5.1 ± 0.4	2.00 ± 0.01	7.8 ± 1.3	1.29 ± 0.49	0.010
	Pt-O-Ce	3.7 ± 0.3	3.11 ± 0.08	-5.6 ± 7.4	10.84 ± 4.12	
**Pt/CeO_2-800**	Pt-O	4.4 ± 0.3	2.00 ± 0.01	7.9 ± 1.9	1.29 ± 0.49	0.021
	Pt-O-Ce	4.2 ± 0.3	3.10 ± 0.07	-2.8 ± 6.8	9.32 ± 3.05	

[a] Coordination number; [b] Bond length; [c] Energy shift; [d] Debye-Waller factor; [e] Goodness-of-fit index.

Supplementary Table 4 Parameters of NH_3-TPD profiles on CeO_2, Pt/CeO_2-550 and Pt/CeO_2-800.

Samples	Peak area ratio (%)			
	**α**	**β**	**γ**	**δ**
**CeO_2**	16	51	33	/
**Pt/CeO_2-550**	4	29	67	/
**Pt/CeO_2-800**	1	20	28	51

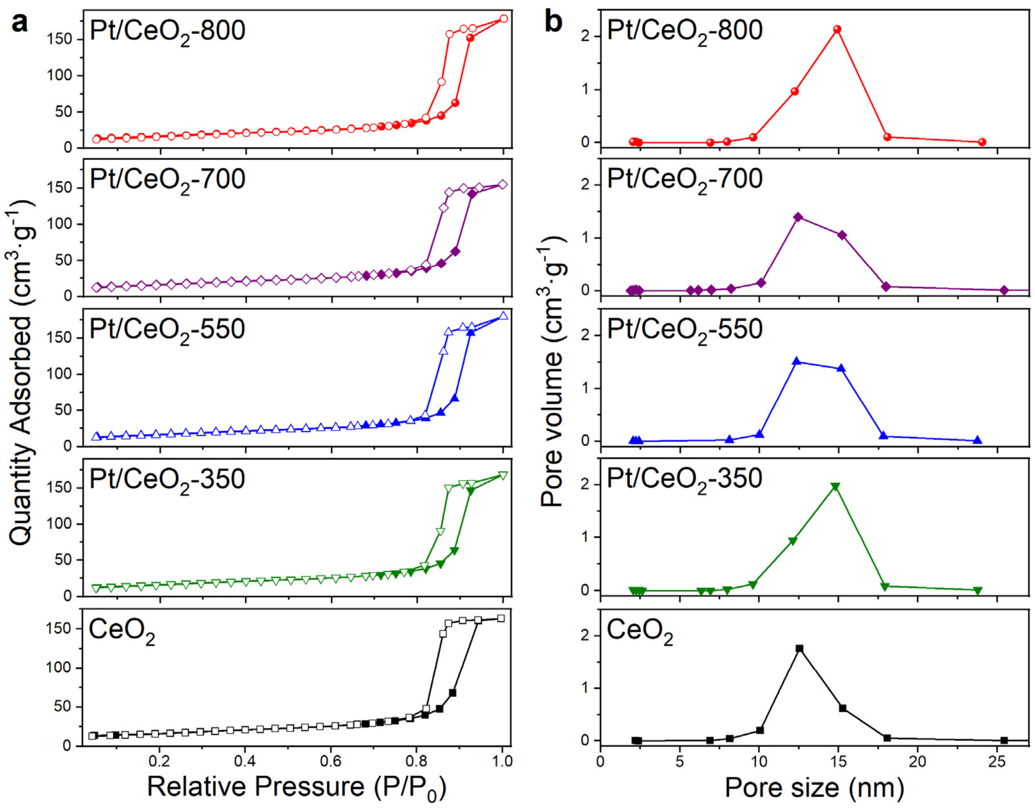

Supplementary Fig. 1 N_2 physisorption results. (a) Nitrogen adsorption-desorption isotherms and **(b)** BJH pore size distribution for CeO_2 and Pt/CeO_2-X (X = 350, 550, 700 and 800).

Notes: CeO_2 and Pt/CeO_2-X catalysts exhibited similar type IV isotherms with H1 hysteresis loops, indicating the mesoporous structure for these samples. Moreover, the pore size distributions on CeO_2 and Pt/CeO_2-X catalysts were almost the same. Taking the similar specific surface areas of these samples into consideration (**Table 1**), it can be concluded that the textual structure of CeO_2 support did not change after the deposition of Pt and the following calcination at different temperatures, owing to the pre-calcination of CeO_2 support at 800 °C for 12 h before the catalyst preparation.

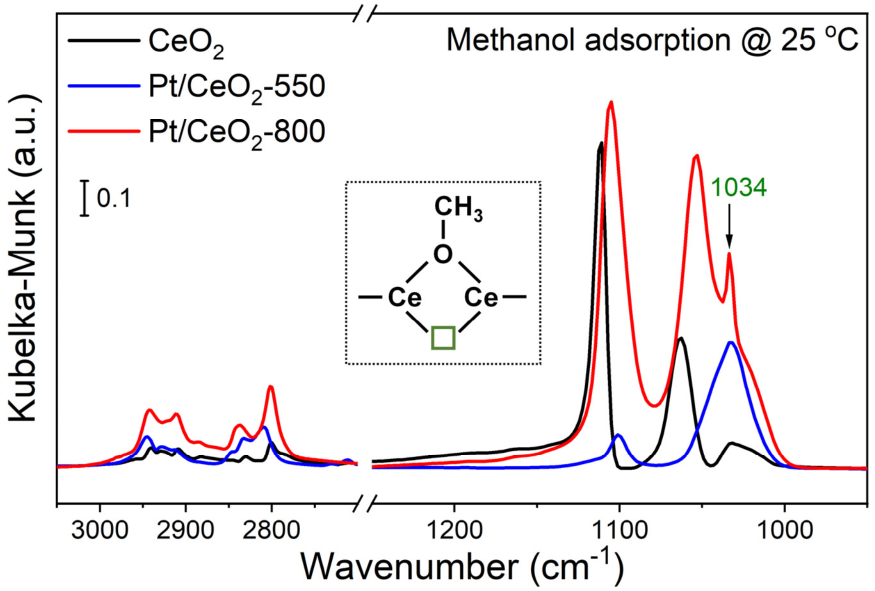

Supplementary Fig. 2 Characterization of surface defects by methanol adsorption. *In situ* DRIFTS of methanol adsorption on CeO_2 support, Pt/CeO_2-550 and Pt/CeO_2-800 at 25 ºC. The olive square indicates the oxygen defects.

Notes: The band at 1034 cm^{-1} could be assigned to bridging methoxy species on two Ce^{4+} cations having an oxygen vacancy.[1] It was clearly observed that the band at 1034 cm^{-1} on Pt/CeO_2-800 showed much higher intensity than that on CeO_2 and Pt/CeO_2-550, suggesting that more surface defects were formed on Pt/CeO_2-800 catalyst. The higher concentration of surface defects on Pt/CeO_2-800 could be induced by the incorporation of Pt ions into the surface lattice of CeO_2 support at high temperature.

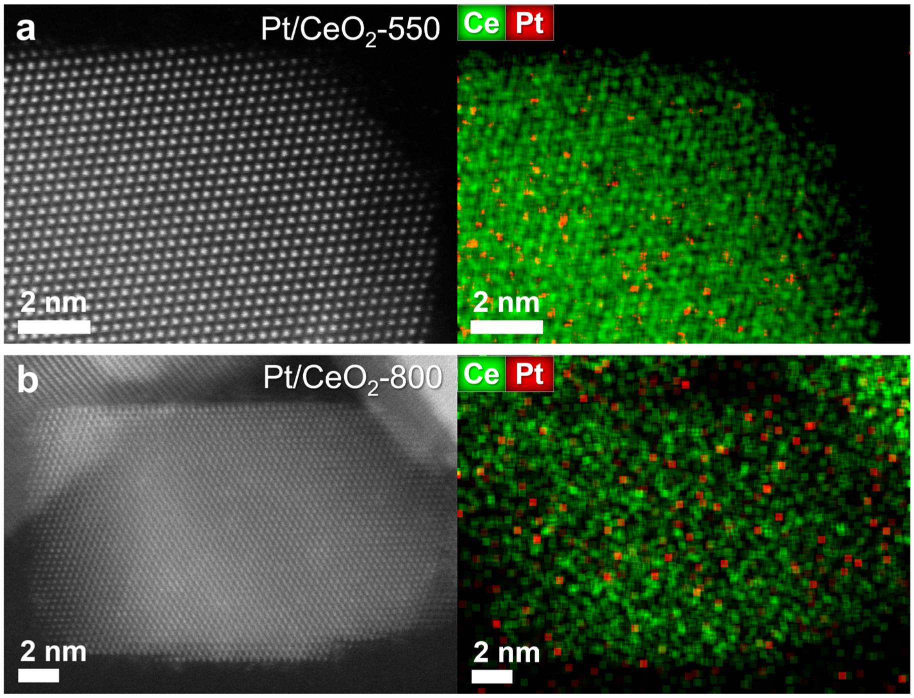

Supplementary Fig. 3 Confirmation of Pt_1 status on CeO_2. AC-HAADF-STEM and corresponding high-resolution EDS mapping images for **(a)** Pt/CeO_2-550 and **(b)** Pt/CeO_2-800 catalysts.

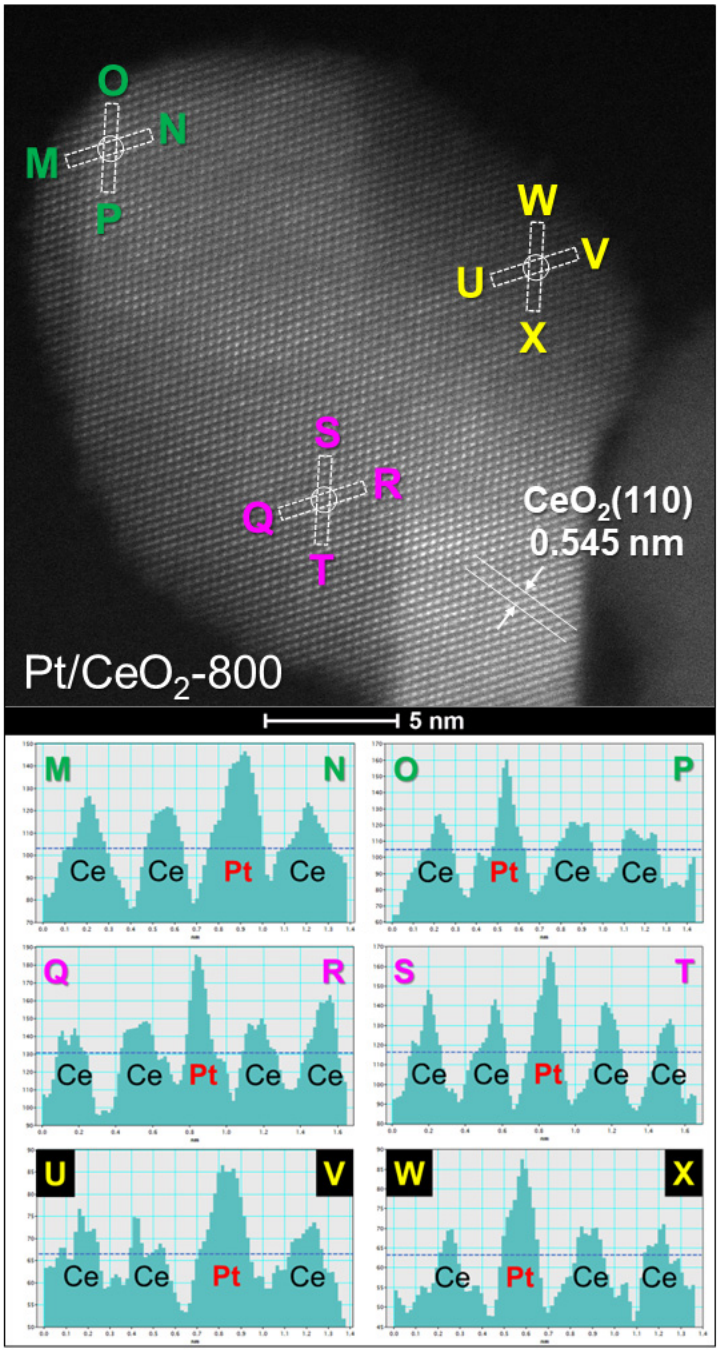

Supplementary Fig. 4 Location of Pt_1 on CeO_2 within Pt/CeO_2-800. AC-HAADF-STEM image (above) and the line profiles (below) of Pt/CeO_2-800 catalyst.

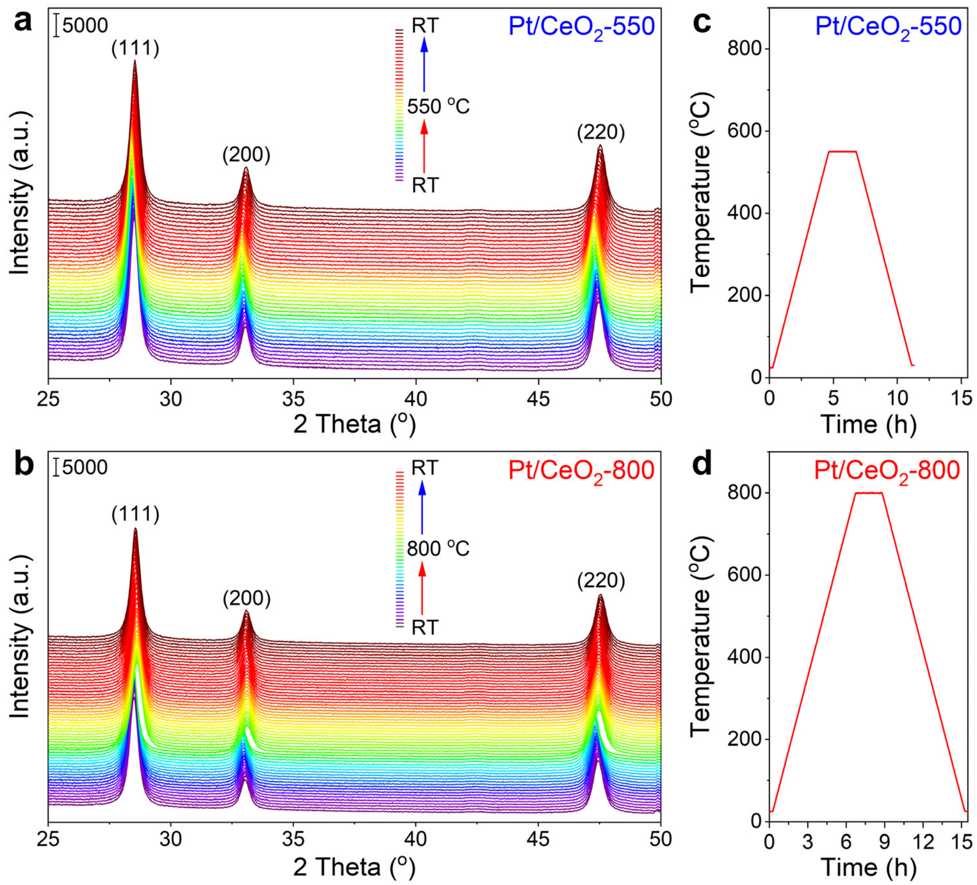

Supplementary Fig. 5 Lattice structure of Pt_1/CeO_2 catalysts. *In situ* XRD patterns for **(a)** Pt/CeO_2-550 and **(b)** Pt/CeO_2-800; Temperature-control programs for **(c)** Pt/CeO_2-550 and **(d)** Pt/CeO_2-800 during the *in situ* XRD experiments.

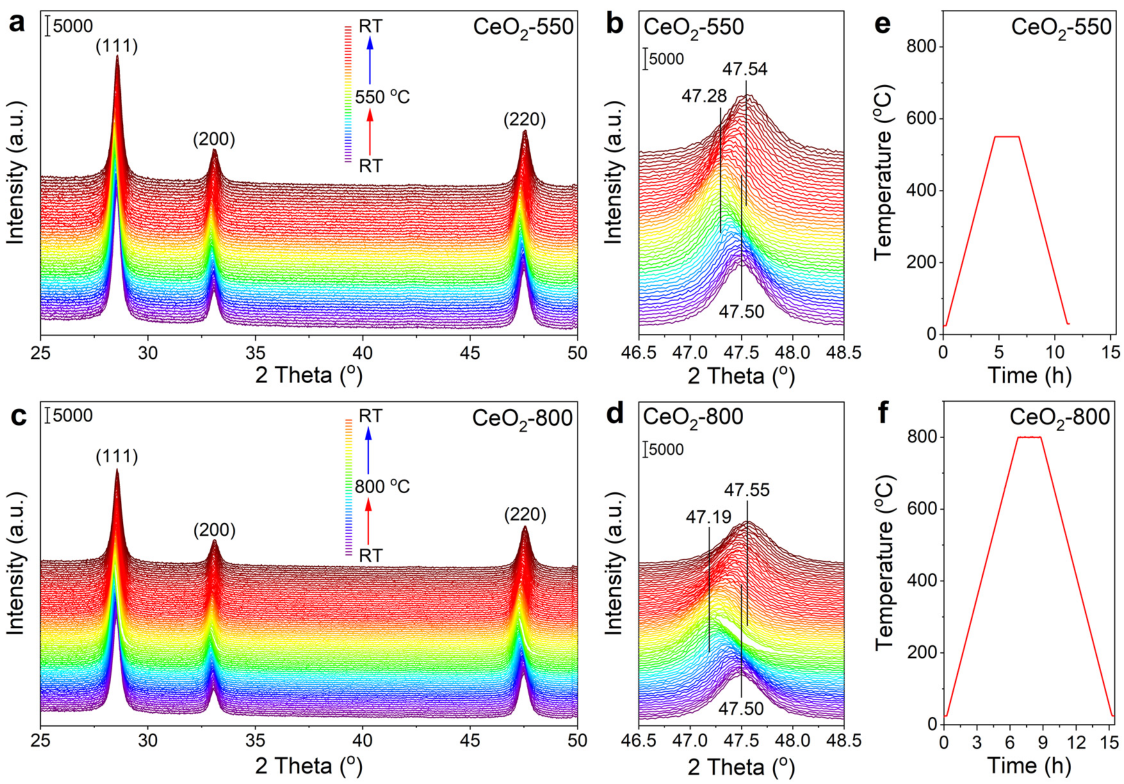

Supplementary Fig. 6 Lattice structure of bare CeO_2 support. *In situ* XRD patterns for bare CeO_2 support **(a, b)** under simulated calcination at 550 °C (labeled as CeO_2-550) and **(c, d)** under simulated calcination at 800 °C (labeled as CeO_2-800); Temperature-control programs during the *in situ* XRD experiments for **(e)** CeO_2-550 and **(f)** CeO_2-800.

Notes: The XRD peaks for CeO_2 showed a similar shift trend during the heating and cooling processes as those for Pt/CeO_2-550 and Pt/CeO_2-800 catalysts. For bare CeO_2 support, the shift of the XRD peaks to higher angles after calcination could be related to the desorption of surface adsorbed H_2O. It is noteworthy that the shift of the XRD peaks on bare CeO_2 after 800 °C calcination (0.05 °) was much smaller than that on Pt/CeO_2-800 (0.09 °), further supporting the viewpoint that Pt^{2+} species with smaller ionic radius should have incorporated into the surface lattice of CeO_2 after 800 °C calcination which resulted in the surface lattice contraction of CeO_2.

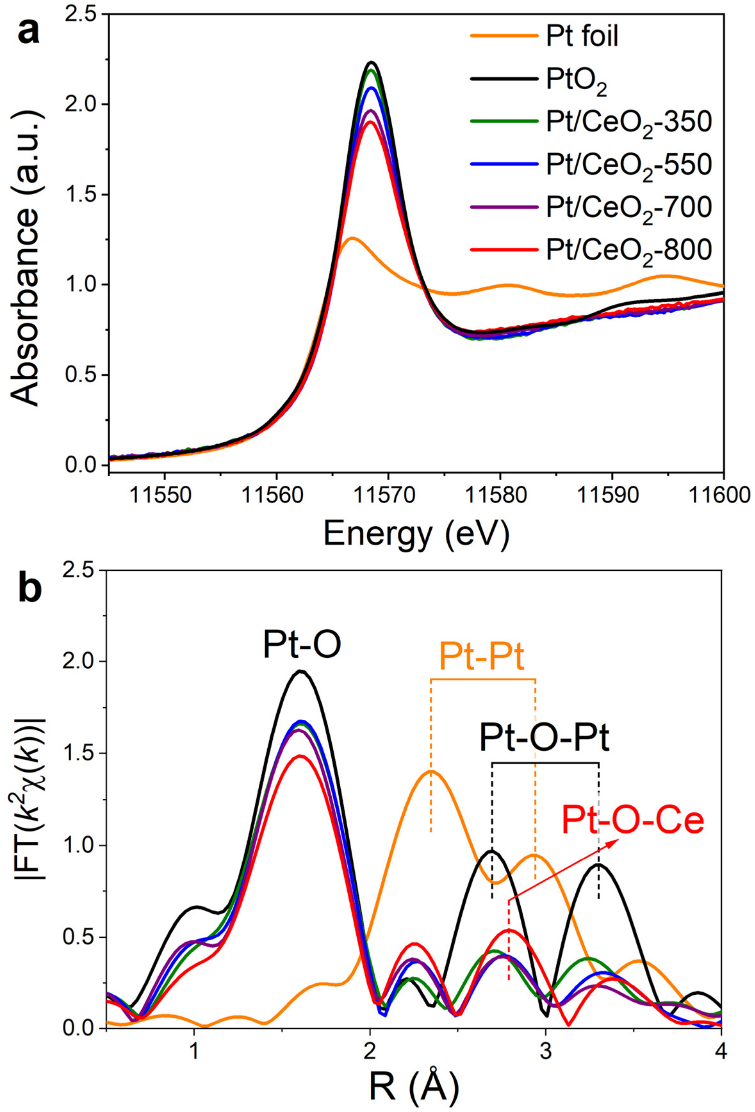

Supplementary Fig. 7 Pt-L_3 edge XAS results. (a) Normalized XANES and **(b)** EXAFS magnitude of the Fourier transformed k^2-weighted $\chi(k)$ data for Pt/CeO_2-X at the Pt-L_3 edge (X = 350, 550, 700 and 800). Pt foil and PtO_2 references were used for comparison.

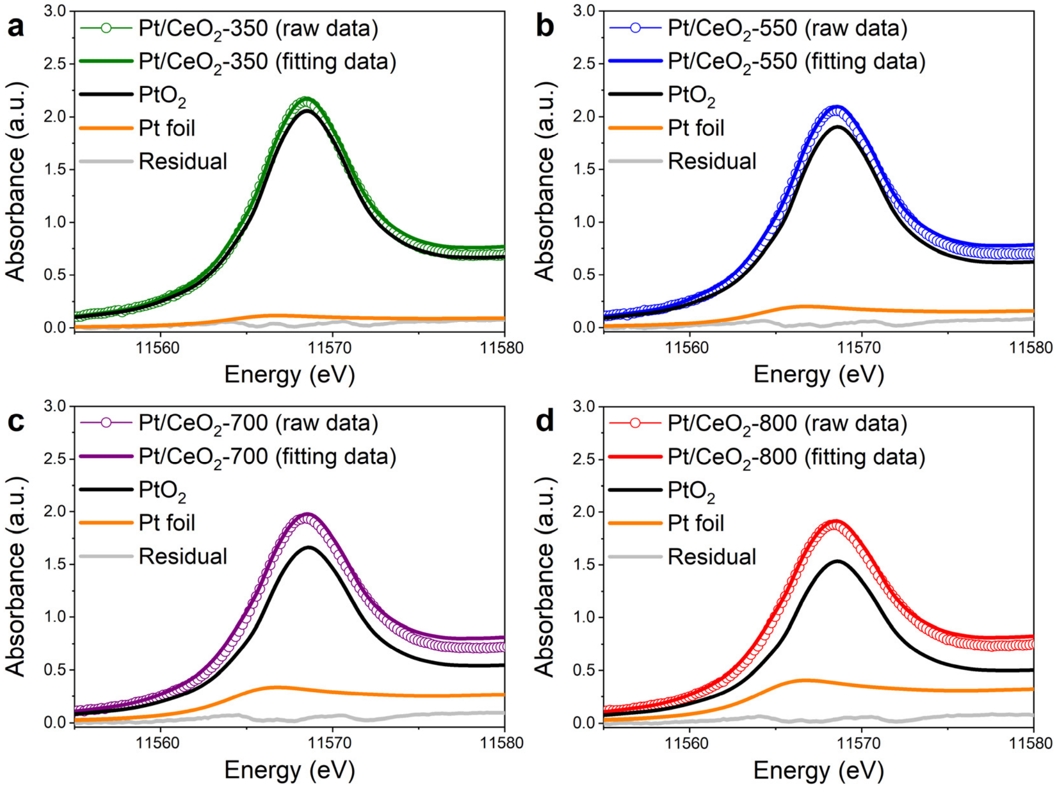

Supplementary Fig. 8 Pt-L_3 edge XANES linear combination fitting results. Linear combination fitting curves of Pt L_3-edge XANES for **(a)** Pt/CeO_2-350, **(b)** Pt/CeO_2-550, **(c)** Pt/CeO_2-700 and **(d)** Pt/CeO_2-800. The detailed fitting results can be found in **Supplementary Table 1**.

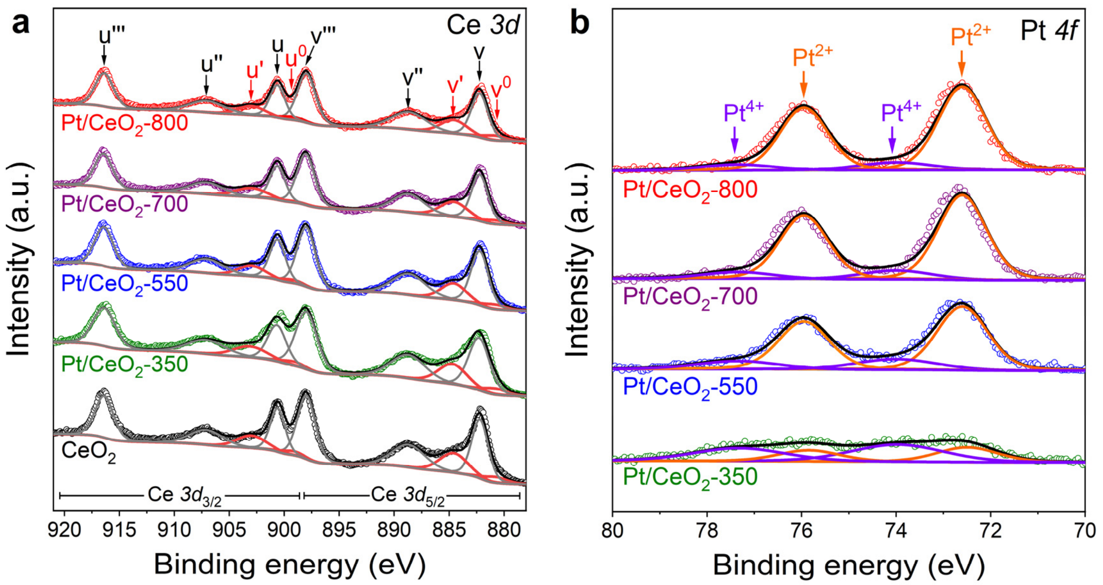

Supplementary Fig. 9 Surface chemical states. (a) XPS of Ce 3d on CeO_2 and Pt/CeO_2-X catalysts; **(b)** XPS of Pt 4f on Pt/CeO_2-X catalysts (X = 350, 550, 700 and 800).

Notes: As already illustrated in the *in situ* DRIFTS of CO adsorption section, the Pt species on Pt/CeO_2-350 were in the form of clusters, and the Pt clusters could disperse into Pt single atoms when the calcination temperature reached 550 °C or higher. That was why the Pt 4f XPS for Pt/CeO_2-550/700/800 showed much higher intensity than that for Pt/CeO_2-350. The surface concentration of Pt atoms (**Supplementary Table 2**) on Pt/CeO_2-550 (0.8%), Pt/CeO_2-700 (1.1%) and Pt/CeO_2-800 (1.1%) could be considered to be comparable. For Pt/CeO_2-800, the Pt atoms were located at the surface substitution sites of Ce (instead of the bulk lattice of CeO_2), therefore the Pt concentration on Pt/CeO_2-800 was observed similar to that on Pt/CeO_2-550/700.

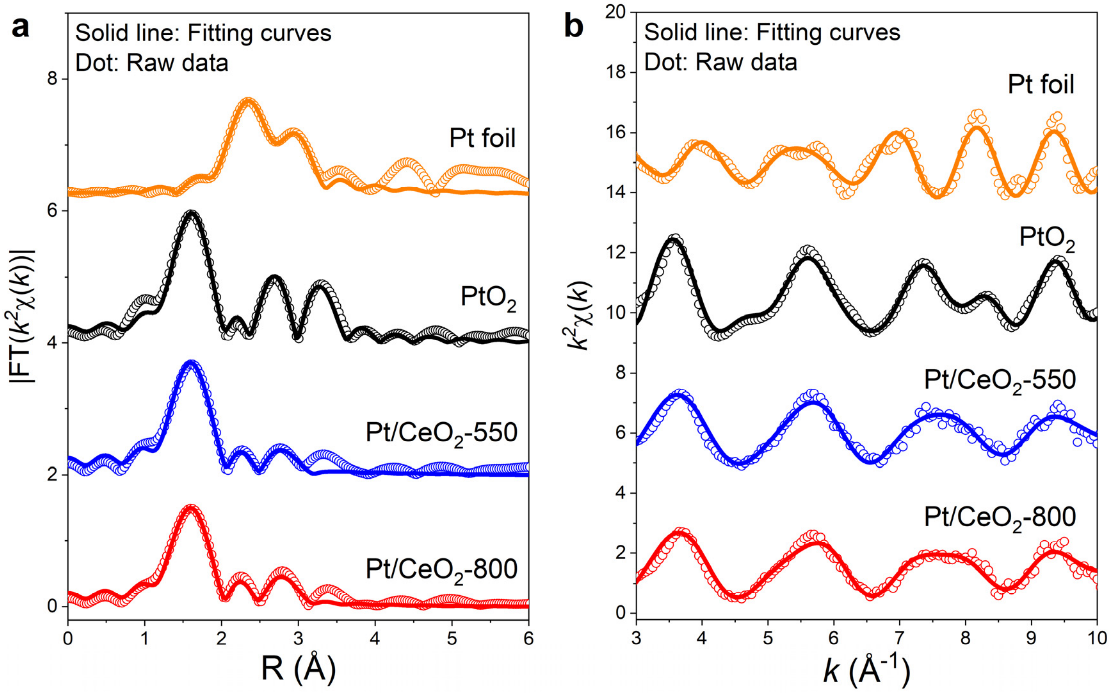

Supplementary Fig. 10 Pt-L$_3$ edge EXAFS curve fitting results. EXAFS fitting curves in **(a)** R-space and **(b)** *k*-space at the Pt-L$_3$ edge. The detailed fitting results can be found in **Supplementary Table 3**.

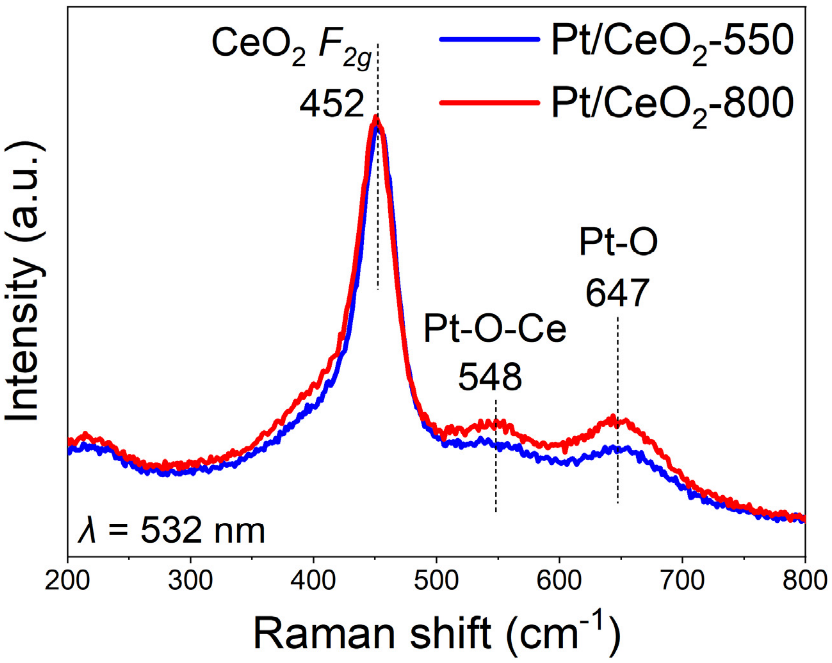

Supplementary Fig. 11 Pt-O and Pt-O-Ce structures characterized by Raman spectra. Raman spectra for Pt/CeO_2-550 and Pt/CeO_2-800.

Notes: Raman spectra for Pt/CeO_2-550 and Pt/CeO_2-800 were collected (**Supplementary Fig. 11**). The sharp band at *ca.* 452 cm^{-1} could be assigned to the triply degenerated F_{2g} mode of fluorite-type CeO_2.[2] The bands at *ca.* 548 and 647 cm^{-1} could be attributed to Pt-O-Ce and Pt-O (in Pt-O-Ce) structures.[3] It was observed that the bands assigned to Pt-O-Ce and Pt-O (in Pt-O-Ce) on Pt/CeO_2-800 showed higher intensity than those on Pt/CeO_2-550, indicating that more Pt-O-Ce linkages were formed on Pt/CeO_2-800, matching well with the EXAFS curve fitting results.

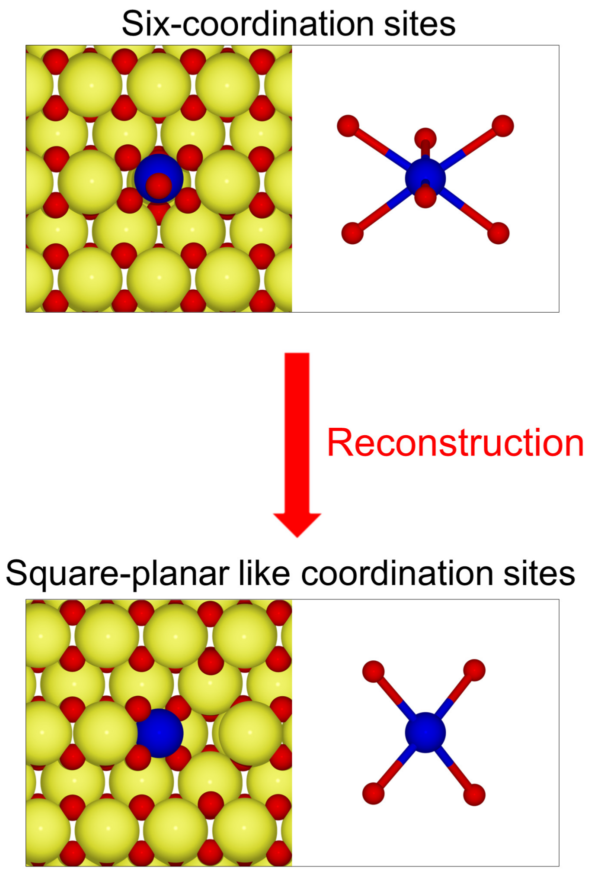

Supplementary Fig. 12 Reconstruction of Pt_1 local coordination structure within Pt/CeO_2-800. The reconstruction of Pt single atoms on CeO_2 (110) from six-coordination sites to square-planar like coordination sites within Pt/CeO_2-800.

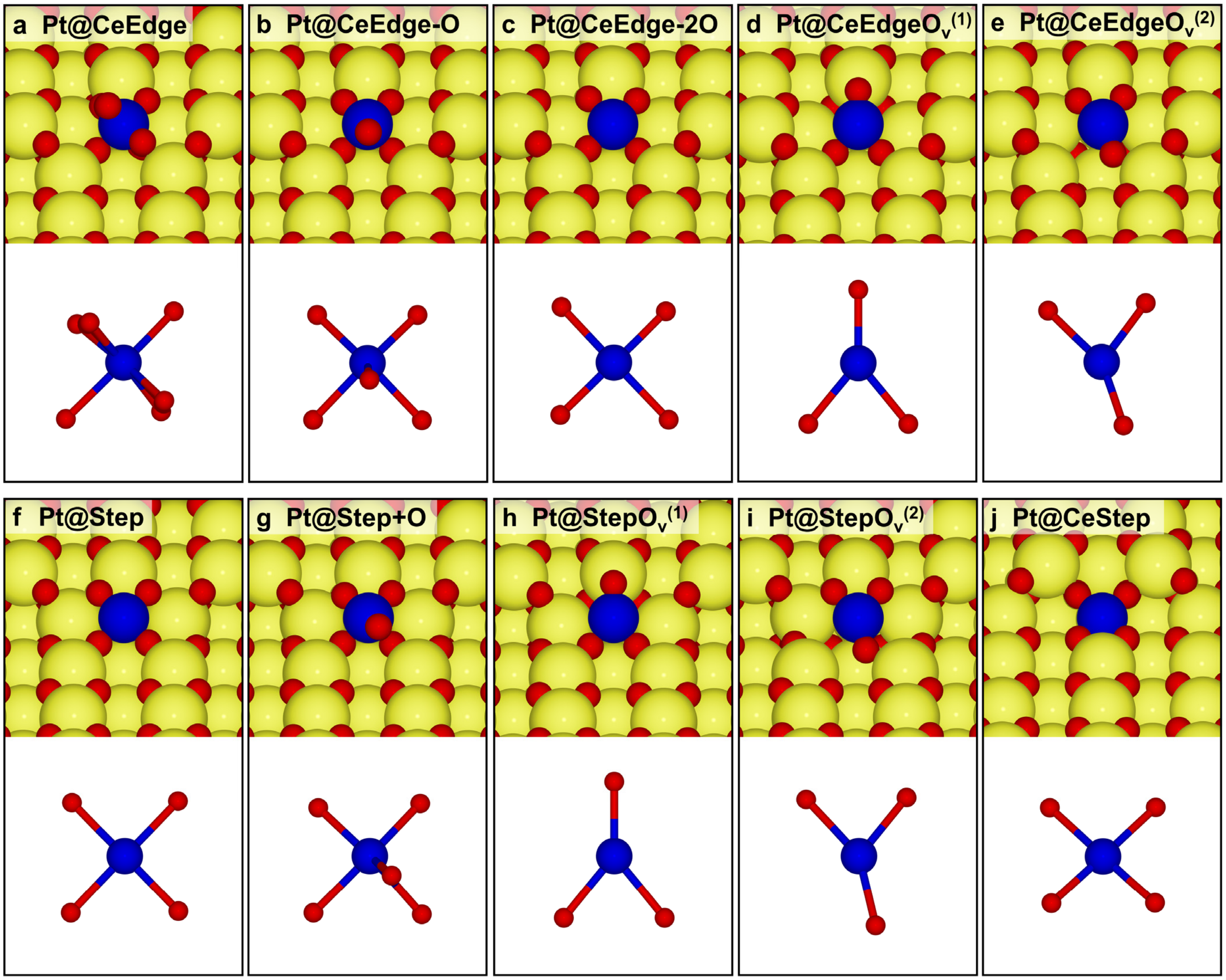

Supplementary Fig. 13 Possible Pt_1/CeO_2 configurations within Pt/CeO_2-550. Possible geometries of Pt single atoms on CeO_2 (110) (top: the top-view of structure; bottom: the coordination of Pt to O) within Pt/CeO_2-550. The name of each configuration is shown on the top left of each panel **(a-j)**.

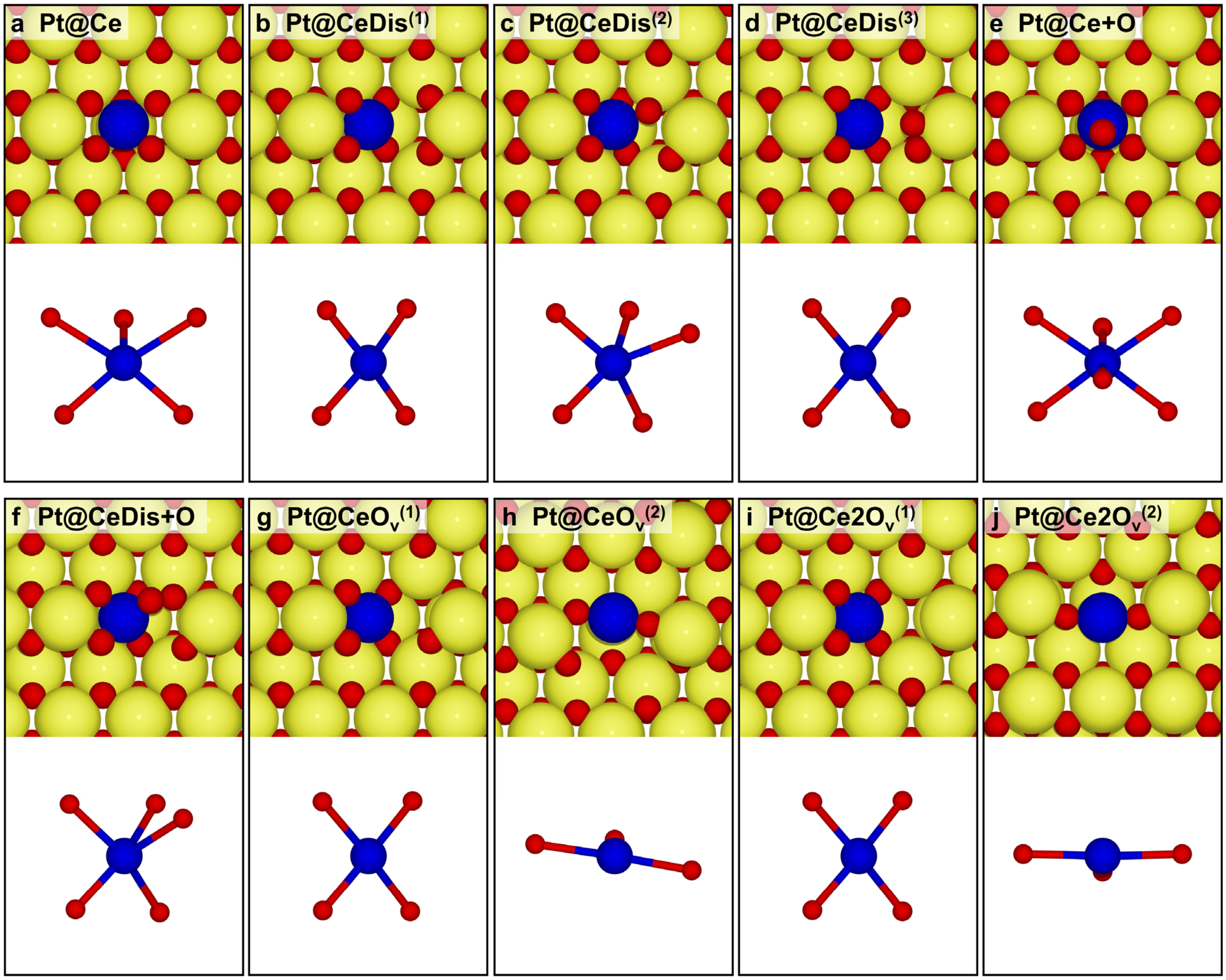

Supplementary Fig. 14 Possible Pt_1/CeO_2 configurations within Pt/CeO_2-800. Possible geometries of Pt single atoms on CeO_2 (110) (top: the top-view of structure; bottom: the coordination of Pt to O) within Pt/CeO_2-800. The name of each configuration is shown on the top left of each panel **(a-j)**.

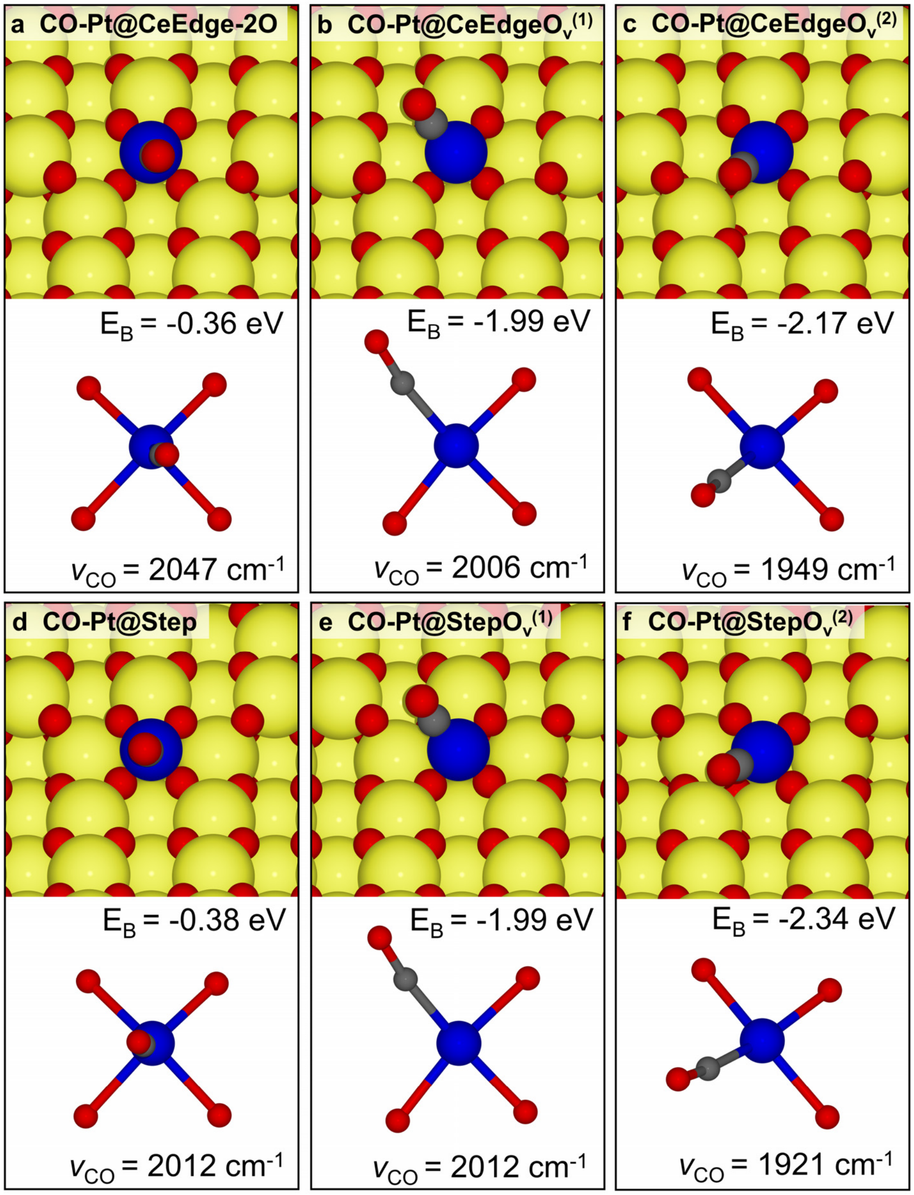

Supplementary Fig. 15 Stretching frequency of CO adsorbed on different Pt_1/CeO_2 configurations within Pt/CeO_2-550. CO adsorption configurations in **(a)** CO-Pt@CeEdge-2O, **(b)** CO-Pt@CeEdgeO$_v$$^{(1)}$, **(c)** CO-Pt@CeEdgeO$_v$$^{(2)}$, **(d)** CO-Pt@Step, **(e)** CO-Pt@StepO$_v$$^{(1)}$, **(f)** CO-Pt@StepO$_v$$^{(2)}$ and corresponding coordination of Pt atoms with the first neighbor O atoms/CO molecules on Pt_1/CeO_2 at edge/step sites of CeO_2 (110) surface for Pt/CeO_2-550 catalyst.

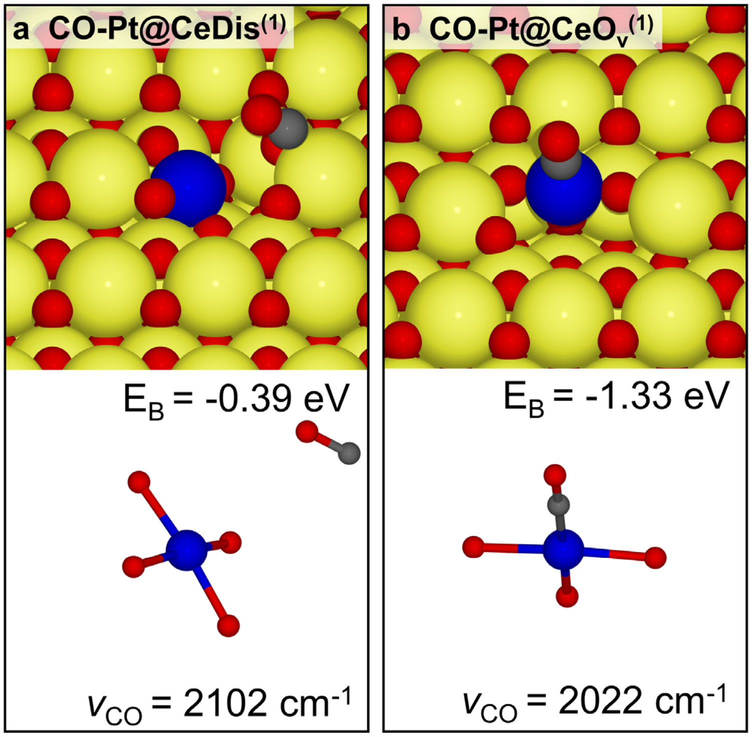

Supplementary Fig. 16 Stretching frequency of CO adsorbed on different Pt_1/CeO_2 configurations within Pt/CeO_2-800. CO adsorption configurations in **(a)** CO-Pt@CeDis$^{(1)}$, **(b)** CO-Pt@$CeO_v^{(1)}$ and corresponding coordination of Pt atoms with the first neighbor O atoms/CO molecules on Pt_1/CeO_2 at terrace sites of CeO_2 (110) surface for Pt/CeO_2-800 catalyst.

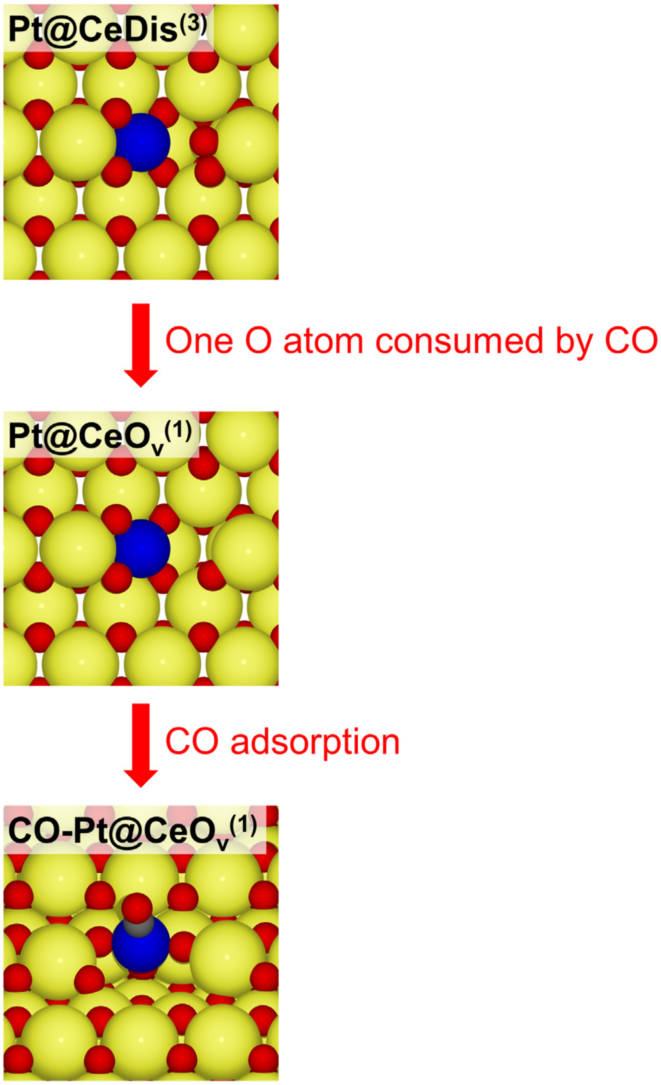

Supplementary Fig. 17 Reconstruction of Pt@CeDis$^{(3)}$ configuration induced by CO adsorption. Scheme of the reconstruction process of Pt@CeDis$^{(3)}$ configuration within Pt/CeO_2-800 under the CO adsorption condition.

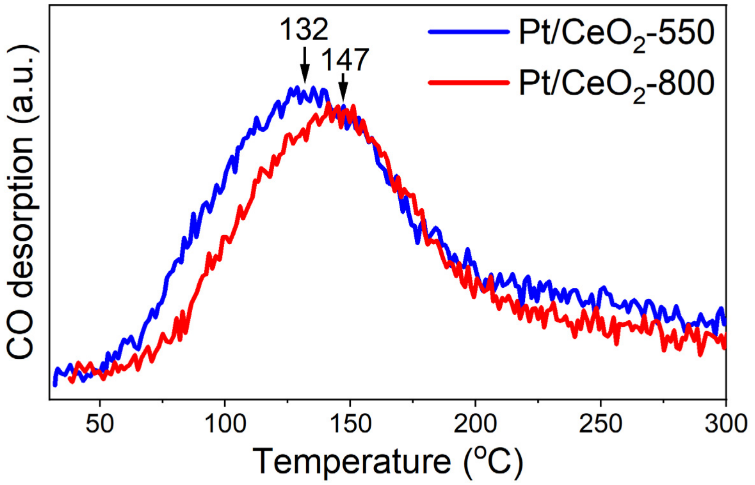

Supplementary Fig. 18 CO adsorption strength determined by CO-TPD. CO-TPD profiles on Pt/CeO_2-550 and Pt/CeO_2-800 catalysts.

Notes: As shown in **Supplementary Fig. 18**, the CO desorption peak on Pt/CeO_2-800 centered at higher temperature (147 °C) than that on Pt/CeO_2-550 (132 °C), suggesting that the adsorption of CO on Pt/CeO_2-800 was indeed stronger than that on Pt/CeO_2-550. The CO adsorption energy was further calculated by Redhead analysis using the equation as below:[4]

$$\frac{E}{RT_p} = \ln\left(\frac{vT_p}{\beta}\right) - 3.64$$

where E is the desorption energy and T_p is the peak desorption temperature. The rate constant was previously determined as $v = 10^{15}$ s^{-1}. For other parameters, β = 10 $K{\cdot}min^{-1}$ = 0.167 $K{\cdot}s^{-1}$, T_p = 405 K (Pt/CeO_2-550) and 420 K (Pt/CeO_2-800).

Therefore, the CO adsorption energy on each catalyst was calculated as: E(Pt/CeO_2-550) = 1.35 eV; E(Pt/CeO_2-800) = 1.40 eV.

In brief summary, the adsorption of CO on Pt/CeO_2-800 was stronger than that on Pt/CeO_2-550, which was consistent with the DFT calculation results.

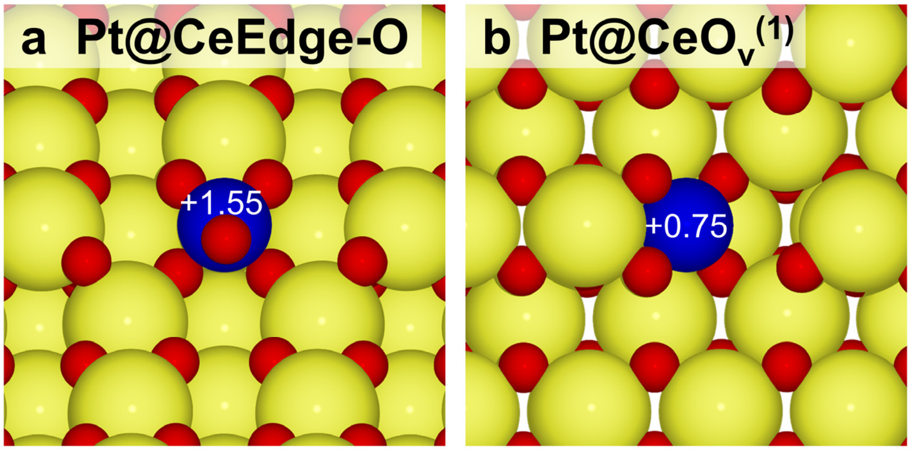

Supplementary Fig. 19 Bader charge analysis for Pt_1 species. Calculated Bader charges for Pt atoms in **(a)** Pt@CeEdge-O (representing Pt/CeO_2-550 catalyst) and **(b)** Pt@$CeO_v^{(1)}$ (representing Pt/CeO_2-800 catalyst).

Notes: The charges of Pt atoms in two configurations, *i.e.*, Pt@CeEdge-O (representing Pt/CeO_2-550 catalyst) and Pt@$CeO_v^{(1)}$ (representing Pt/CeO_2-800 catalyst), were evaluated using the Bader analysis, based on the method reported in literature.[5, 6]

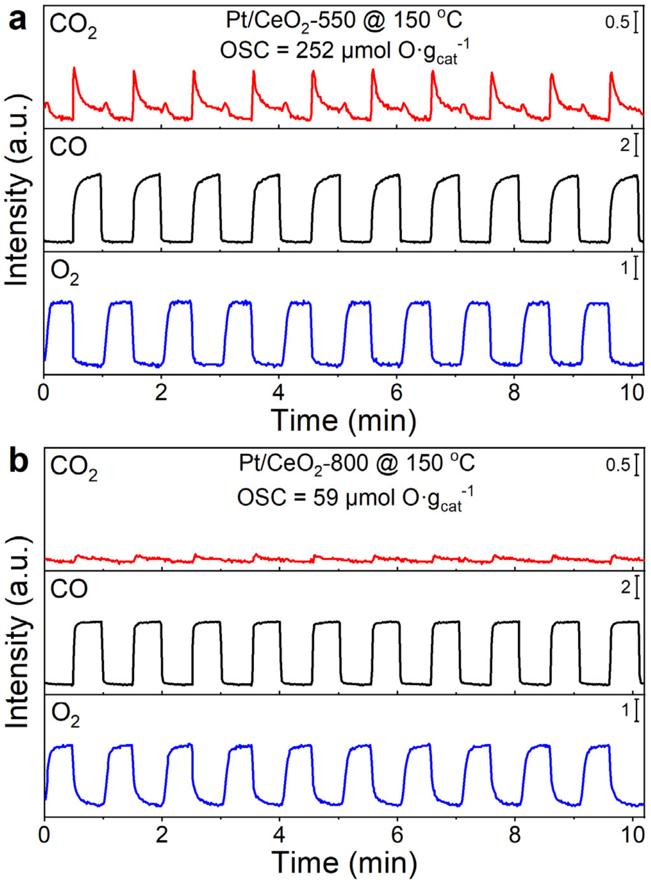

Supplementary Fig. 20 Dynamic OSC testing results. Signals of CO, O_2, and CO_2 monitored by an online mass spectrometer in the dynamic OSC test for **(a)** Pt/CeO_2-550 and **(b)** Pt/CeO_2-800.

Notes: The dynamic oxygen storage capacity (OSC) test was performed at 150 °C, in which the catalysts were exposed to CO/O_2 cyclic flow and the CO_2 formation was monitored online (**Supplementary Fig. 20**). Much higher concentration of CO_2 was formed over Pt/CeO_2-550 catalyst during the testing, suggesting the much higher dynamic OSC function of Pt/CeO_2-550 (252 μmol O·g_{cat}^{-1}) than that of Pt/CeO_2-800 (59 μmol O·g_{cat}^{-1}). O_2 could be better activated and transferred on Pt/CeO_2-550 than on Pt/CeO_2-800, which was one of the main reasons for the much higher CO oxidation activity on Pt/CeO_2-550 catalyst.

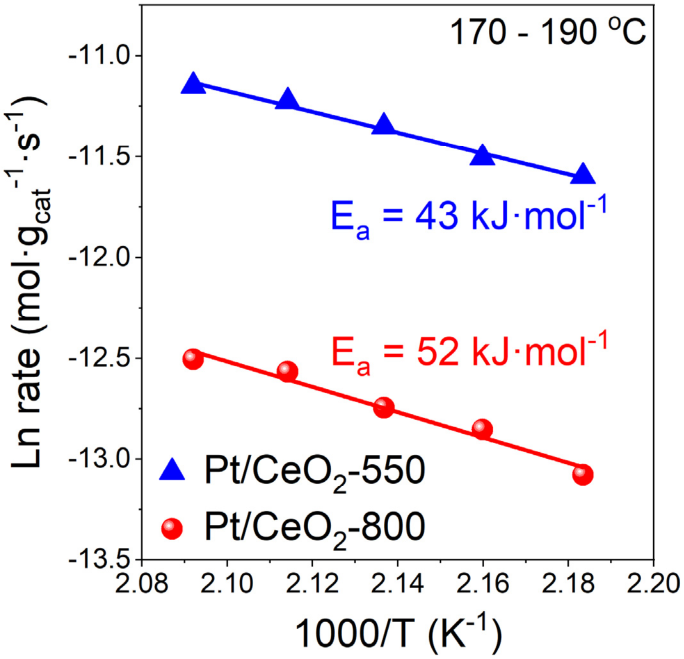

Supplementary Fig. 21 Apparent activation energy (E_a) for CO oxidation on Pt_1/CeO_2 catalysts. Arrhenius plots and E_a values for CO oxidation on Pt/CeO_2-550 and Pt/CeO_2-800. [CO] = [O_2] = 1 vol%, balanced with Ar. The WHSVs for CO oxidation testing on Pt/CeO_2-550 and Pt/CeO_2-800 were controlled at 800,000 and 400,000 $mL \cdot g_{cat}^{-1} \cdot h^{-1}$, respectively.

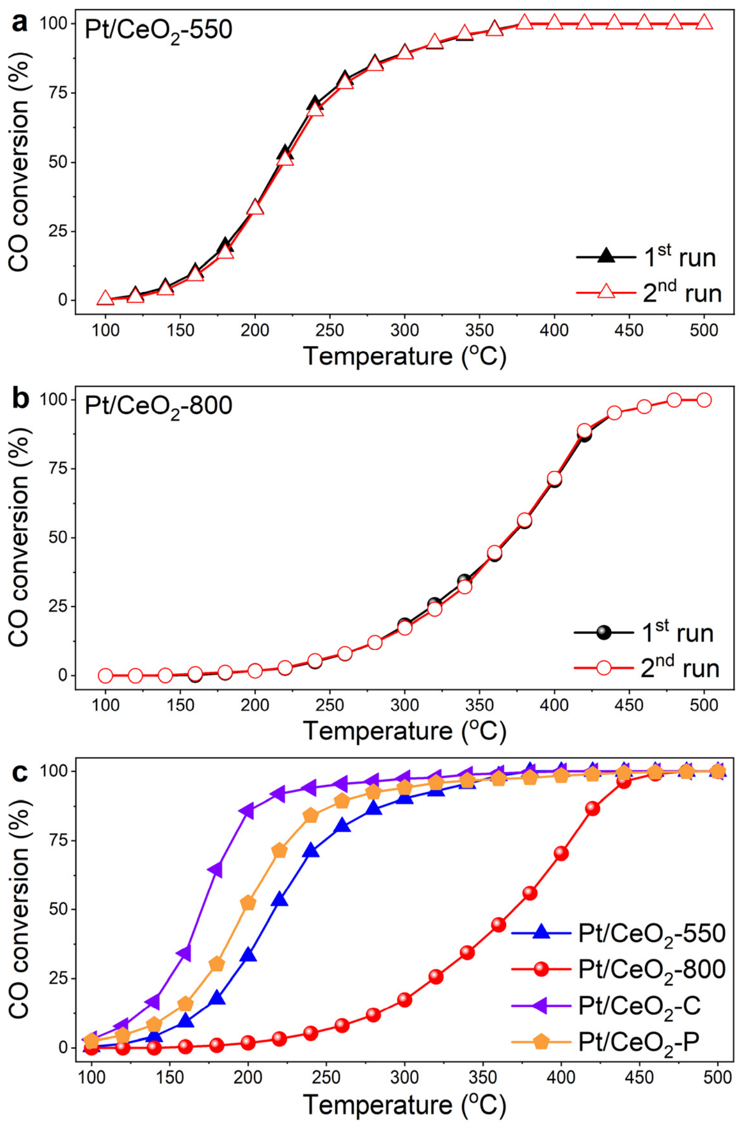

Supplementary Fig. 22 Stability of Pt_1/CeO_2 catalysts under CO oxidation atmosphere and CO oxidation activity on Pt/CeO_2 catalysts with different Pt dispersion states. CO conversions on **(a)** Pt/CeO_2-550 and **(b)** Pt/CeO_2-800 during the two-round CO oxidation activity test; **(c)** CO oxidation activity on Pt/CeO_2-550, Pt/CeO_2-800, Pt/CeO_2-C and Pt/CeO_2-P. Reaction condition: [CO] = [O_2] = 1 vol%, balanced with Ar, WHSV = 400,000 $mL\cdot g_{cat}^{-1}\cdot h^{-1}$.

Notes: To evaluate the stability of Pt_1/CeO_2 catalysts in this work, a two-round CO oxidation activity test (from RT to 500 °C) was conducted on both Pt/CeO_2-550 and Pt/CeO_2-800. As shown in **Supplementary**

Fig. 22a-b, the CO oxidation activity on Pt/CeO_2-550 and Pt/CeO_2-800 showed almost no change during the two rounds of tests, suggesting that the Pt_1/CeO_2 catalysts prepared in this work showed high stability under the CO oxidation conditions.

To compare the CO oxidation activity on Pt single atom, cluster and nanoparticle catalysts, we prepared CeO_2 supported Pt clusters (Pt/CeO_2-C) and nanoparticles (Pt/CeO_2-P) as well. The Pt clusters (1-2 nm) were synthesized according to the previously reported method.[7] The solution of Pt colloidal (4-6 nm) was used as the precursor of Pt nanoparticles. As shown in **Supplementary Fig. 22c**, the Pt cluster catalyst showed higher CO oxidation activity than the Pt nanoparticle catalyst. Both Pt clusters and Pt nanoparticles performed better CO oxidation performance than Pt single atoms on Pt/CeO_2-550 and Pt/CeO_2-800, which could also suggest that the Pt single atoms did not aggregate into Pt clusters or nanoparticles during the two-round CO oxidation activity test.

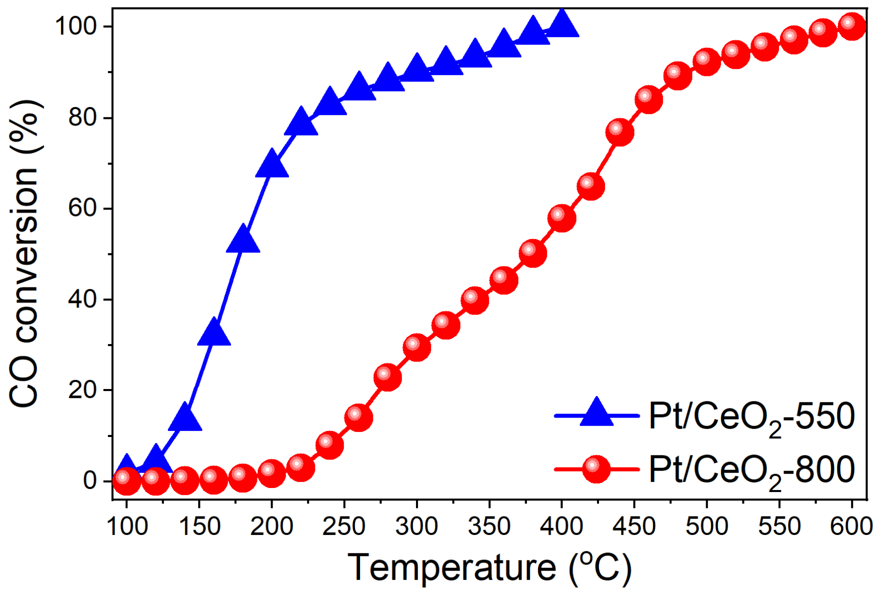

Supplementary Fig. 23 CO oxidation activity evaluated under wet condition. CO oxidation performance on Pt/CeO_2-550 and Pt/CeO_2-800 catalysts under wet condition: [CO] = 1%, [O_2] = 1%, [H_2O] = 5%, balanced with Ar, WHSV = 400,000 mL·g_{cat}^{-1}·h^{-1}.

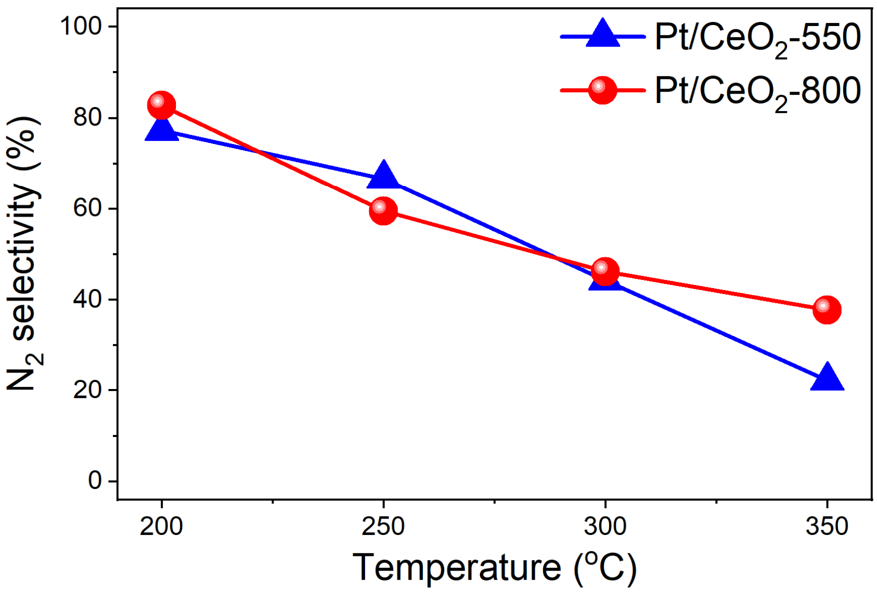

Supplementary Fig. 24 N_2 selectivity in NH_3 oxidation reaction on Pt_1/CeO_2 catalysts. The N_2 selectivity in NH_3 oxidation reaction on Pt/CeO_2-550 and Pt/CeO_2-800 (Reaction condition: [NH_3] = 500 ppm, [O_2] = 5 %, balanced with Ar, WHSV = 200,000 mL·g_{cat}^{-1}·h^{-1}).

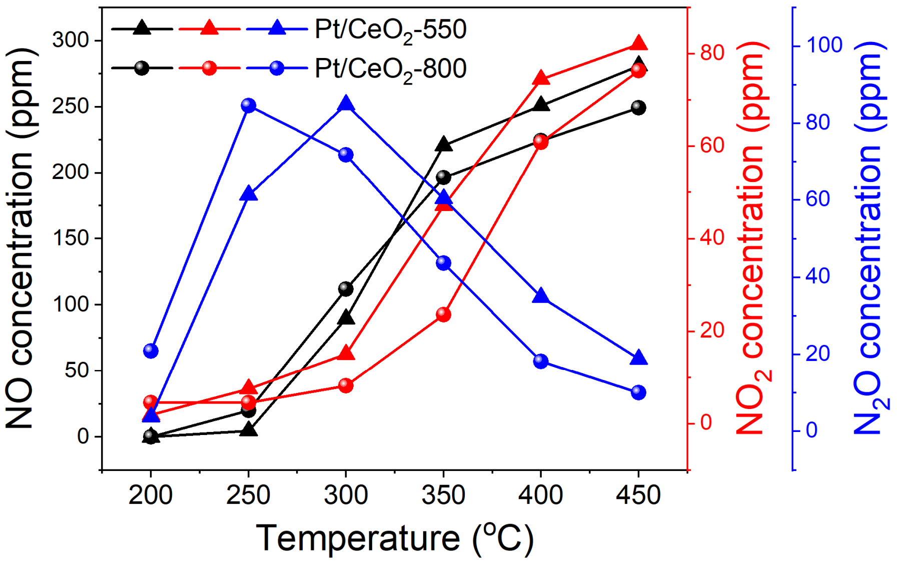

Supplementary Fig. 25 Formation of non-selective oxidation products in NH_3 oxidation reaction. The formation of NO, NO_2 and N_2O as a function of temperature in NH_3 oxidation reaction on Pt/CeO_2-550 and Pt/CeO_2-800 catalysts.

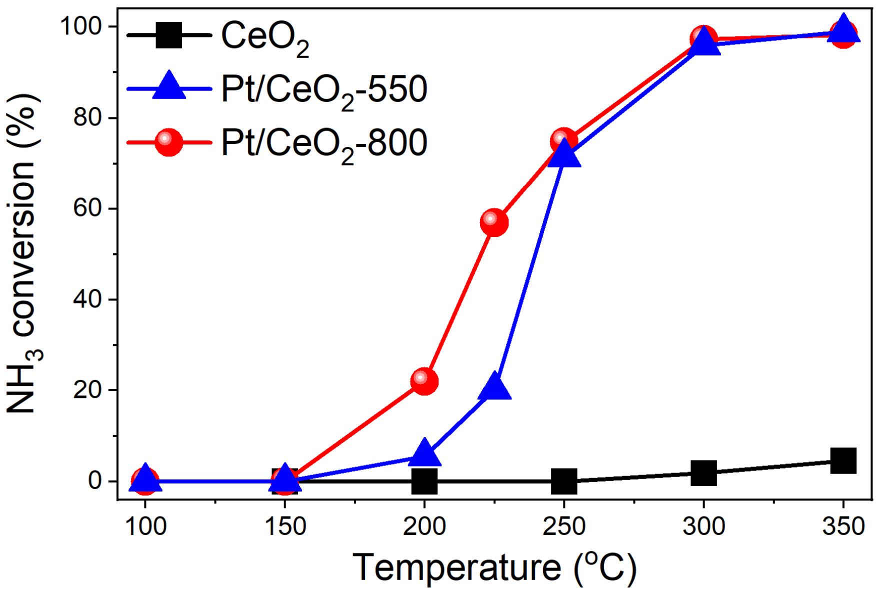

Supplementary Fig. 26 NH_3 oxidation activity evaluated under wet condition. NH_3 oxidation performance on Pt/CeO_2-550 and Pt/CeO_2-800 catalysts as well as CeO_2 support under wet condition: [NH_3] = 500 ppm, [O_2] = 5%, [H_2O] = 5%, balanced with Ar, WHSV = 200,000 mL·g_{cat}^{-1}·h^{-1}.

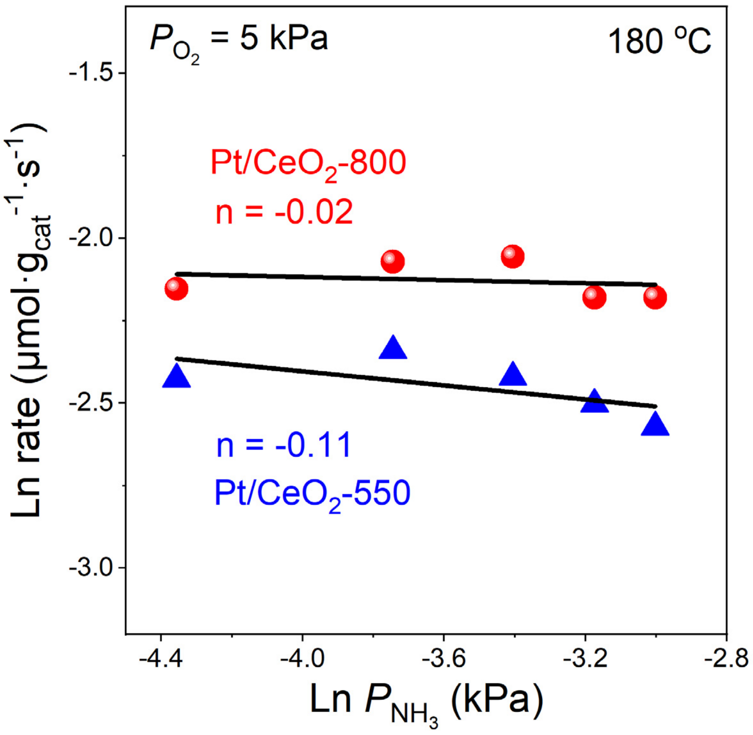

Supplementary Fig. 27 Kinetics study of NH_3 oxidation on Pt_1/CeO_2 catalysts. The NH_3 reaction order in NH_3 oxidation reaction on Pt/CeO_2-550 and Pt/CeO_2-800 catalysts. To avoid heat transfer effects, the reaction rates were determined with the NH_3 conversions below 15%. Reaction temperature was fixed at 180 ºC. Reaction conditions: [NH_3] = 100 to 500 ppm, [O_2] = 5 vol%, balanced with Ar, WHSV = 600,000 mL·g_{cat}^{-1}·h^{-1}.

Notes: Since the concentration of O_2 is usually much higher than that of NH_3 in typical NH_3 oxidation reaction, we did not measure the O_2 reaction order in this study.

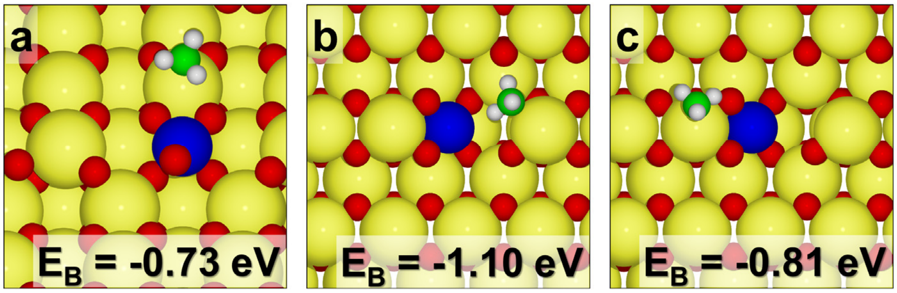

Supplementary Fig. 28 NH_3 adsorption by DFT simulations. NH_3 adsorption energy on **(a)** Pt@CeEdge-O and **(b, c)** Pt@$CeO_v^{(1)}$ configurations.

Notes: It was found that NH_3 could not adsorb on the Pt atoms but could adsorb on the nearby Ce atoms for both Pt@CeEdge-O and Pt@$CeO_v^{(1)}$ configurations. On Pt@$CeO_v^{(1)}$ configuration, the NH_3 adsorption energy on Ce atoms with (**Supplementary Fig. 28b**) and without (**Supplementary Fig. 28c**) oxygen vacancy nearby was calculated as -1.10 eV and -0.81 eV, respectively. Since NH_3 was found to bind stronger on the Ce sites with oxygen vacancy nearby, the DFT calculation on NH_3 oxidation mechanism over Pt@$CeO_v^{(1)}$ structure (representing Pt/CeO_2-800 catalyst) was started from this configuration (**Supplementary Fig. 28b**).

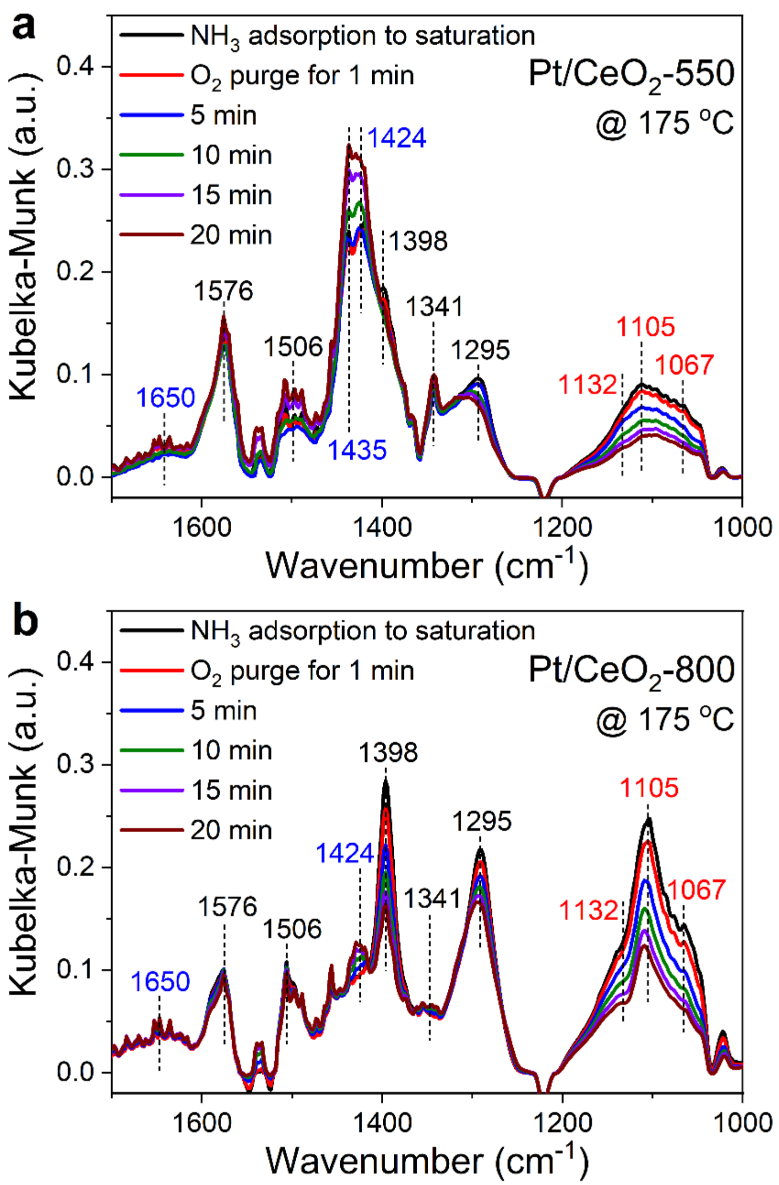

Supplementary Fig. 29 Reactivity of adsorbed NH_3 species on Pt_1/CeO_2 catalysts. *In situ* DRIFTS of O_2 reacting with pre-adsorbed NH_3 on **(a)** Pt/CeO_2-550 and **(b)** Pt/CeO_2-800 at 175 ºC (before introducing O_2 to the DRIFTS cell, the catalysts saturated with NH_3 were purged by Ar to remove physically/weakly adsorbed NH_3).

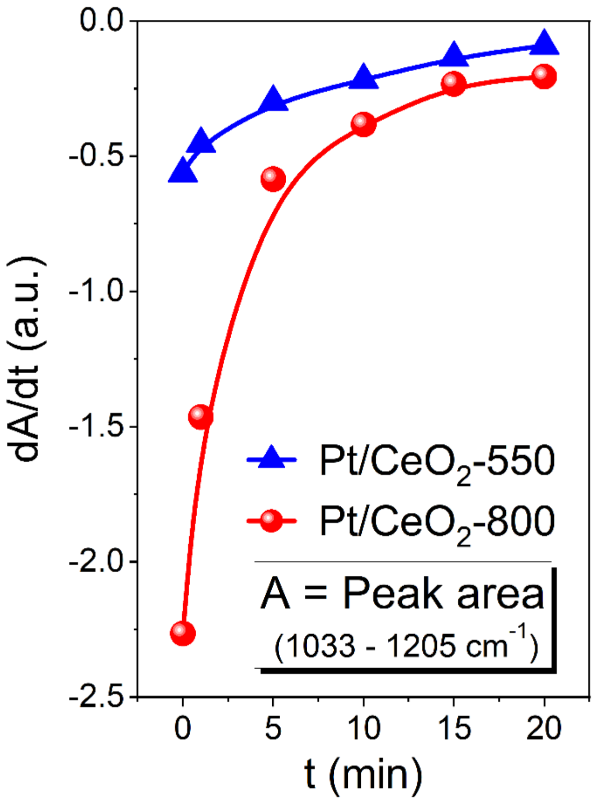

Supplementary Fig. 30 Relative reaction rate of pre-adsorbed NH_3 species reacting with O_2. First-order derivative of peak area (1033-1205 cm^{-1}) in **Supplementary Fig. 29** with respect to reaction time.

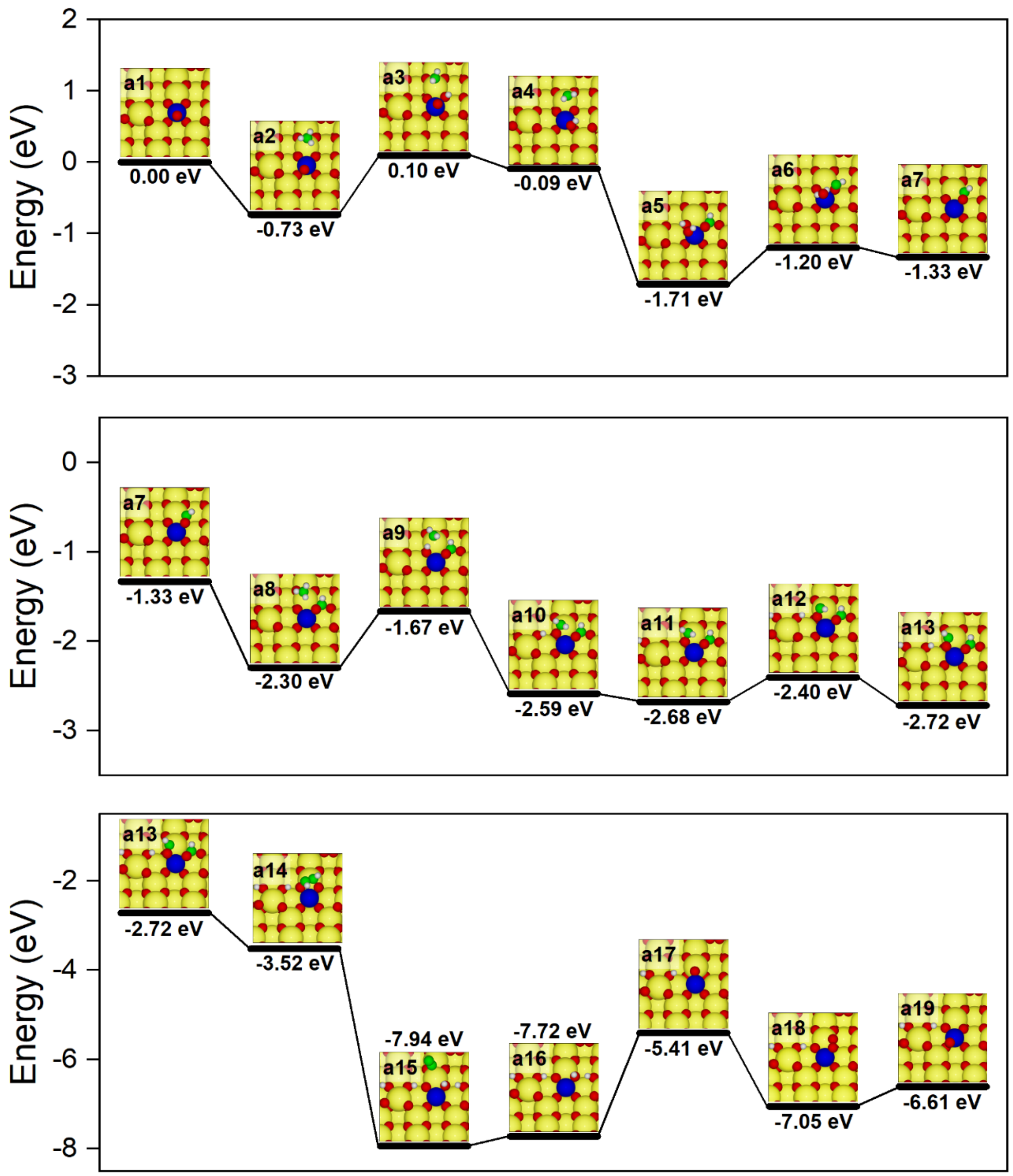

Supplementary Fig. 31 NH_3 oxidation mechanism on Pt/CeO_2-550. Proposed mechanism for NH_3 oxidation on Pt@CeEdge-O structure (dominant configuration on Pt/CeO_2-550). The potential energy was calculated with respect to NH_3 and O_2 in gas phase. Large yellow, medium blue, small red, green, and white balls represented Ce, Pt, O, N, and H atoms, respectively.

Notes: The proposed mechanism for NH_3 oxidation on Pt@CeEdge-O is shown in **Supplementary Fig. 31**. The oxidation of NH_3 on Pt@CeEdge-O started with the adsorption of NH_3 on the surface Ce atom adjacent to the Pt atom with an adsorption energy of -0.73 eV (a2), followed by the dissociation of N-H bond to form -NH_2 species and H atom that bound to an O atom adjacent to the Pt atom (a3). This process was endothermic with a reaction energy of 0.83 eV. Then, the H atom transferred to O atom on top of Pt to form -OH group (a4) with a reaction energy of -0.19 eV. The -NH species and H atom generated by the dissociation of another N-H bond could be coordinated to the O atoms adjacent to the Pt atom (a5). This process was exothermic with a reaction energy of -1.80 eV. Then, the H atom could combine with the -OH group adsorbed on top of Pt atom to form H_2O molecule (a6). This process was endothermic with a reaction energy of 0.51 eV. Subsequently, the formed H_2O molecule could desorb from the surface and at the same time the -NHO species reconfigured itself to bind on both Pt and surface Ce atom (a7) with a reaction energy of -0.13 eV. Then, the second NH_3 was found to co-adsorb on the Ce atom (a8) with a reaction energy of -0.97 eV, followed by an endothermic N-H bond dissociation process with a reaction energy of 0.63 eV, resulting in the formation of -NH_2 group and H atom that adsorbed on O atom (a9). The -NH_2 group could then push the H atom to a nearby O atom to form -NH_2O group (a10), with a reaction energy of -0.92 eV. Interestingly, the H atom was found to prefer to move to the O atom far away from Pt atom (a11). Then, the N-H bond in -NH_2O could further dissociate to form configuration a12, with a reaction energy of 0.28 eV, which could then reconfigure itself into configuration a13 in which the N atoms of two -NH groups were coordinated to O atoms nearby the Pt atom. Moreover, these two -NH groups were found to prefer to combine together to from N_2H_2 species (a14), with a reaction energy of -0.80 eV. From this configuration, once one N-H bond dissociated, the second N-H bond was found to dissociate spontaneously as well, thus generating a N_2 molecule and two H atoms on the surface (a15). The reaction energy from a14 to a15 was -4.42 eV, indicating the high instability of N_2H_2 species and the

preference of N_2 formation. This N_2 molecule was found to desorb with a desorption energy of 0.22 eV (a16). Afterwards, the H atom and -OH group combined to form the second H_2O molecule, which could desorb from the surface thus creating one oxygen vacancy (a17). It is noteworthy that the desorption of this H_2O molecule was highly endothermic with a reaction energy of 2.31 eV, indicating that the formation and desorption of the second H_2O molecule on Pt@CeEdge-O was the rate limiting step in this mechanism. The oxygen vacancy was then filled by the adsorption and dissociation of O_2 (a18 and a19). The adsorption of O_2 was an exothermic process (-1.64 eV) while the dissociation of O_2 was endothermic with a reaction energy of 0.44 eV.

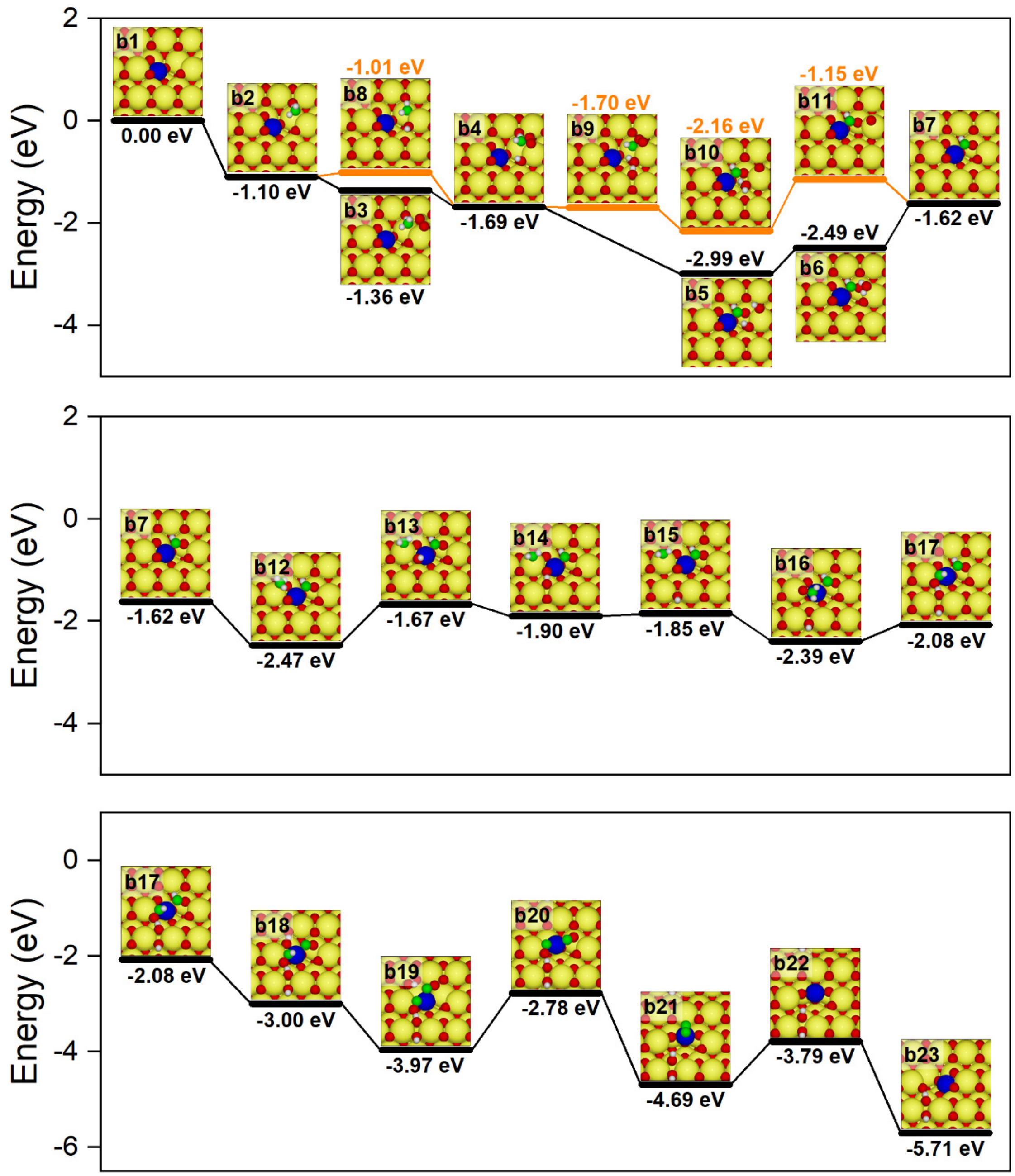

Supplementary Fig. 32 NH_3 oxidation mechanism on Pt/CeO_2-800. Proposed mechanism for NH_3 oxidation on Pt@CeO_v[1] structure (dominant configuration on Pt/CeO_2-800). The potential energy was calculated with respect to NH_3 and O_2 in gas phase. Large yellow, medium blue, small red, green, and white balls represented Ce, Pt, O, N, and H atoms, respectively.

Notes: The proposed mechanism for NH_3 oxidation on Pt@$CeO_v^{(1)}$ is shown in **Supplementary Fig. 32**. After the adsorption of NH_3 (b2, E_B = -1.10 eV), O_2 was adsorbed on the same Ce atom to form co-adsorption configuration with a binding energy of -1.36 eV (b3), followed by the dissociation of one N-H bond of NH_3. This formed H atom could combine with a surface O atom to form configuration b4. Then, the adsorbed O_2 could dissociate and react with -NH_2 species to form -NHO and -OH group (b5), with a reaction energy of -1.30 eV. Subsequently, the formed OH group combined with the H atom to form a H_2O molecule (b6), which could then desorb from the surface (b7).

Besides, it was found that the -NHO species on the Pt@$CeO_v^{(1)}$ structure could be formed through another pathway. In details, one N-H bond of the adsorbed NH_3 molecule could dissociate to form -NH_2 and -OH group (b8), followed by the adsorption of O_2 molecule (b4). Then, another N-H bond could dissociate (b9) and react with the dissociated O_2 to form -NH groups and H_2O (b10). H_2O could then desorb from the surface, leaving an oxygen vacancy (b11). The oxygen vacancy could be filled by O atom from the dissociated O_2 to form configuration b7.

Similar to what was observed on Pt@CeEdge-O structure, the second NH_3 was found to adsorb at the Ce site nearby the Pt atom with a binding energy of -0.85 eV (b12), followed by a series of N-H bond dissociations and H atom diffusions to form adsorbed H_2O species and two -NHO groups (b12 → b17). The N-H bond of one -NHO group could further dissociate to form -NO and -OH group (b18), with a reaction energy of -0.92 eV. Then, the N-H bond of -NHO group could dissociate, and the resulting H atom could combine with the -OH group to form the second H_2O molecule (b19) with a reaction energy of -0.97 eV. The second H_2O molecule was found to desorb with a desorption energy of 1.19 eV, which was an endothermic process, leaving one oxygen vacancy on the surface (b20). The two N atoms from -NO groups could combine to form N_2 adsorbed on the Pt atom (b21), and this process was exothermic with a reaction energy of -1.91 eV. The desorption of N_2 (b21 → b22) was an endothermic process with a

reaction energy of 0.90 eV. After N_2 desorption, a Pt-O_3 structure was formed ($Pt@CeO_v^{(2)}$), which could then reconstruct into $Pt@CeO_v^{(1)}$ configuration. In this proposed reaction pathway, the endothermic process for second H_2O desorption (b19 → b20) was the rate limiting step.

References

1. Wu Z. *et al.* Probing the surface sites of CeO_2 nanocrystals with well-defined surface planes via methanol adsorption and desorption. *ACS Catal.* **2**, 2224-2234 (2012).
2. Loridant S. Raman spectroscopy as a powerful tool to characterize ceria-based catalysts. *Catal. Today* **373**, 98-111 (2021).
3. Brogan MS. *et al.* Raman spectroscopic study of the Pt-CeO_2 interaction in the Pt/Al_2O_3-CeO_2 catalyst. *J. Chem. Soc., Faraday trans.* **90**, 1461-1466 (1994).
4. Thang HV. *et al.* Nature of stable single atom Pt catalysts dispersed on anatase TiO_2. *J. Catal.* **367**, 104-114 (2018).
5. R.F.W. Bader. Atoms in Molecules: A Quantum Theory. Oxford University Press, New York; 1994.
6. W. Tang, E. Sanville, and G. Henkelman. A grid-based Bader analysis algorithm without lattice bias. *J. Phys. Condens. Matter* **21**, 084204 (2009).
7. Quinson J. *et al.* Investigating particle size effects in catalysis by applying a size-controlled and surfactant-free synthesis of colloidal nanoparticles in alkaline ethylene glycol: Case study of the oxygen reduction reaction on Pt. *ACS Catal.* **8**, 6627-6635 (2018).
